# Ferric quinate (QPLEX) interacts with the major outer membrane protein (MOMP) of *Campylobacter jejuni* and enters through the porin channel into the periplasmic space

**DOI:** 10.1016/j.csbj.2022.09.032

**Published:** 2022-09-24

**Authors:** Jennifer C. Okoye, Jeddidiah Bellamy-Carter, Neil J. Oldham, Neil J. Oldfield, Jafar Mahdavi, Panos Soultanas

**Affiliations:** a School of Chemistry, Biodiscovery Institute, University of Nottingham, University Park NG7 2RD, United Kingdom; b School of Chemistry, University of Nottingham, University Park NG7 2RD, United Kingdom; c School of Life Sciences, University of Nottingham, University Park NG7 2RD, United Kingdom

## Abstract

Ferric chelates like ferric tyrosinate (TYPLEX) and the closely related ferric quinate (QPLEX) are structural mimics of bacterial siderophores. TYPLEX has been trialled as a feed additive in farming of commercial broilers, reducing Campylobacter loads by 2–3 log_10_ and leading to faster growth and better feed consumption. These ferric chelates offer a good alternative feed additive to antibiotics helping to reduce the indiscriminate use of preventative antibiotics in broiler farming to control Campylobacter infections. In this study, we show that QPLEX binds to the Major Outer Membrane Protein (MOMP) of *C. jejuni* NCTC11168. MOMP is an essential and abundant outer membrane porin on the surface of the bacteria, acting as an adhesin to help establish infection by mediating attachment of *C. jejuni* onto the gut epithelium of broilers and establish infection. Using carbene footprinting, we map the MOMP-QPLEX interaction and show by complementary *in silico* docking that QPLEX enters the porin channel through interactions at the extracellular face, translocates down the channel through a dipole transverse electric field towards the opposite end and is released into the periplasm at the intracellular face of MOMP. Our studies suggest a potential mechanism for the non-antibiotic anti-Campylobacter activity of these ferric chelates.

## Graphical abstract

**Figure:**
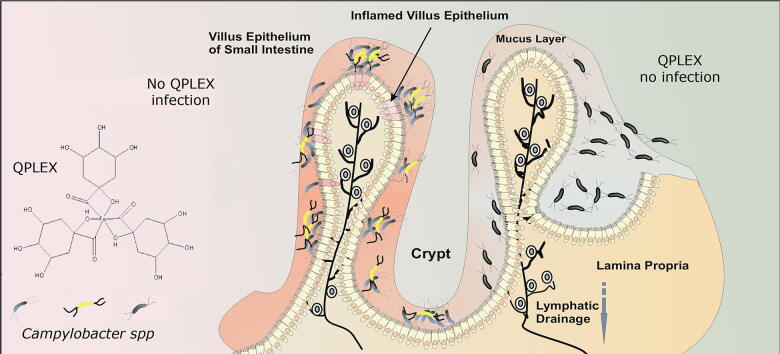

## 1 Introduction

*Campylobacter jejuni* is one of the key global causes of human gastrointestinal disease with significant costs to healthcare systems around the world [1]. The consumption of contaminated and undercooked poultry meat is the major source of human infection, with colonization of gut epithelia ensuing from as low as 500–800 bacteria causing acute gastroenteritis with symptoms, such as inflammation, abdominal pain, fever and diarrhea, lasting up to two weeks [2]. The disease can be fatal to immunocompromised patients [3] and can cause long term serious, life-changing effects, including Guillain-Barré and Miller-Fischer syndromes [4], chronic intestinal disease [5] and arthritis [6]. Successful colonization of the human intestinal mucosa takes place through attachment to a range of histo-blood group antigens (BgAgs) on the gut epithelia, including several related fucose-containing BgAgs [7], as well as core-I, core-II, H-II, Le^b^, Le^y^ and Le^x^ [8]. ABO and Lewis BgAgs are associated with susceptibility to several bacterial infections, including *Helicobacter pylori* (a close relative of *C. jejuni*) infections mediated by interactions of BabA and SabA with BgAgs [9], [10], [11], [12] and *C. jejuni* infections mediated by the Major Outer Membrane Protein MOMP and the flagellar protein FlaA interactions with BgAgs [8], [13].

MOMP is a trimeric 18-stranded antiparallel β-barrel porin with an elliptical shape similar to BabA [14], [15]. The protein has a central pore size and conductivity intermediate between *E. coli* OmpC and OmpF [14]. *O*-glycosylation of *C. jejuni* NCTC11168 MOMP at Thr268 appears to affect its interaction with BgAgs, with a single T268G substitution significantly reducing binding to BgAgs and colonization of chicken gut epithelia [8]. Deletion of *pseD*, encoding a putative PseAm transferase, also resulted in significant reduction in the binding of *C. jejuni* NCTC11168 to BgAgs, suggesting its involvement in MOMP *O*-glycosylation [8]. Most *O-*glycans attached to the *Campylobacter* flagellum are derivatives of pseudaminic acid (Pse) or legionaminic acid (Leg), which are C9 sugars related to sialic acids [16].

Inhibition of *C. jejuni* adhesion to chicken gut epithelia by metal complexes, such as ferric tyrosinate (TYPLEX) used as feed additives, demonstrated significant benefits against *C. jejuni* infections, including 2–3 log_10_ reduction in *C. jejuni* loads [17], [18], [19]. The mechanism of action of TYPLEX is not known but, as it inhibits biofilm formation and attachment to the chicken gut epithelia, it was postulated to act via MOMP [17]. TYPLEX typifies ferric chelates, an emerging group of siderophore mimics, with anti-biofilm activity.

Here, we use ferric quinate (QPLEX), a ferric chelate equivalent to TYPLEX but more soluble than TYPLEX, to show that these ferric complexes bind to MOMP and internalize into the periplasm. The MOMP-QPLEX interaction was mapped using carbene footprinting and, in combination with *in silico* molecular docking, we show that QPLEX initially binds to the extracellular loop region of MOMP and then translocates through the porin channel into the periplasmic space of *C. jejuni*. Better understanding of the mechanism of action of ferric chelates will aid in their development as feed additives in animal farming to replace indiscriminate use of preventative antibiotics in our efforts to combat antibiotic resistance.

## 2 Results

### 2.1 Optimisation of carbene footprinting conditions

The interaction of MOMP with QPLEX was investigated using carbene footprinting [20], [21]. The use of photoactivatable aryl diazirine for efficient footprinting of soluble proteins is well established. The amphipathic nature of the probe may also cause it to interact and footprint outer transmembrane regions of micelle-solubilised proteins like MOMP [21]. To explore the extent to which detergent was being labelled, and thus which detergent would be better for downstream footprinting experiments, a comparative analysis was carried out between poly(ethylene glycol) octyl ether (C_8_E_n_ (n = 2 to 9)) (octyl-POE) detergent and Octyl-beta-Glucoside (OG) detergents (Fig. S1). The results showed that in the presence of octyl*-*POE, there was 25 % product yield, whilst in the presence of OG, the product yield was 20 %. Thus, for increased labelling of outer membrane proteins such as MOMP, octyl*-*POE should be preferred as the detergent for carbene footprinting experiments.

Optimisation of labelling was carried out at different MOMP:probe concentrations using a combination of proteases (trypsin and chymotrypsin) and changing the irradiation time to obtain maximum labelling across the surface of MOMP. Peptides were analysed with PepFoot to discern the extent of labelling [22]. To calculate the fractional modification of carbene labelling, individual data files were batch-processed in PepFoot. Several MOMP peptides were labelled by carbene (≥labelled) whilst some peptides did not show significant labelling (<labelled).

Combining data from digestions with trypsin and chymotrypsin in parallel experiments resulted in 55 % coverage for total peptides that were identified but not labelled (Fig. S2A) and 69 % coverage for total peptides that were identified and labelled (Fig. S2B). The total coverage of identified peptides across both trypsin and chymotrypsin digestions was 95 % (Fig. S2C). It is important to consider all identified peptides, as changes in protein conformation upon ligand binding may cause increased or decreased labelling of different surface regions depending on changes in exposure as these regions become more concealed or exposed.

### 2.2 Mapping the interactions of MOMP with QPLEX

**Fig. 1:**
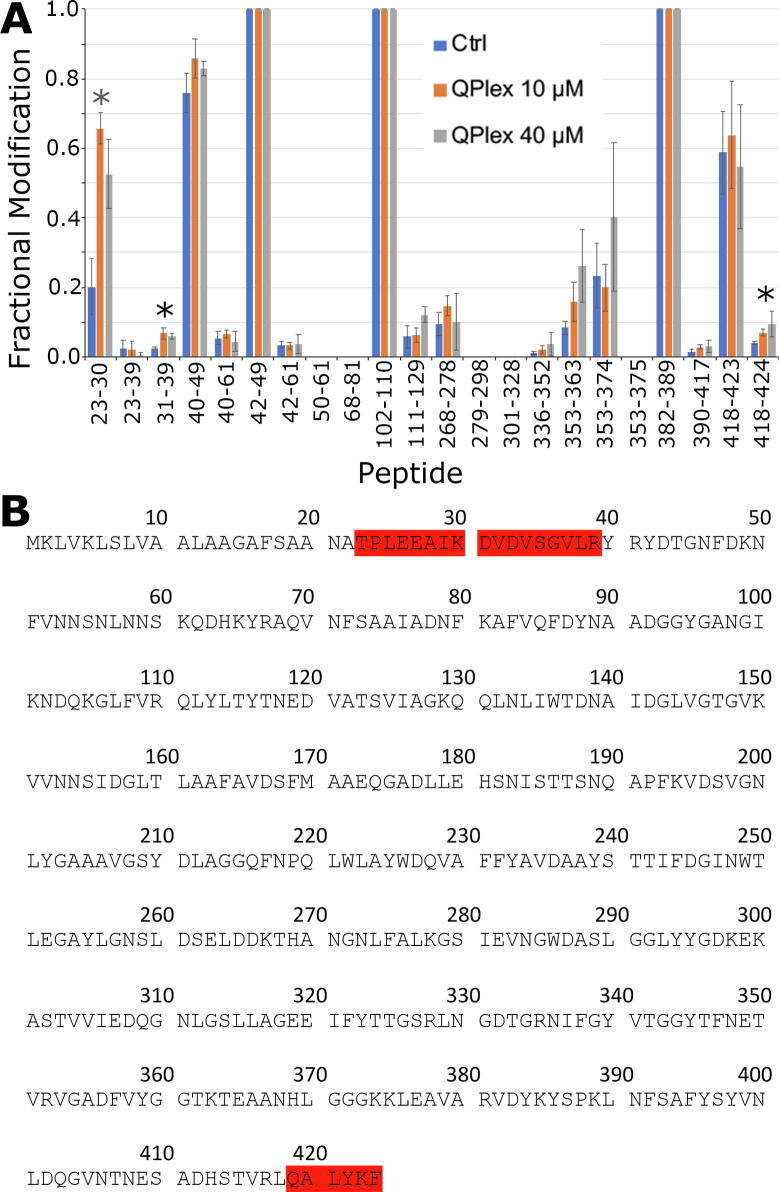
Carbene labelling of MOMP in the absence and presence of QPLEX: Trypsin digests. **A.** Fractional modification of tryptic MOMP peptides after carbene labelling in the absence and presence of 10 and 40 µM QPLEX. Each peptide was given a peptide ID consistent with the position of the amino acid residues in the protein monomer. Blue bars indicate fractional modification of the native peptide in the absence of QPLEX whilst orange and grey bars indicate fractional modification of peptides in the presence of 10 and 40 μM QPLEX. All experiments were performed in triplicate and the mean peptide fractional modification was included with standard error bars about the mean. The significance level of the statistical difference between the control peptide and peptide in presence of QPLEX is shown by asterisks (*); * = p ≤ 0.05, analysed by paired student *t*-test. **B.** Peptides where a significant change in labelling occurs have been mapped onto the sequence of MOMP in red. (For interpretation of the references to colour in this figure legend, the reader is referred to the web version of this article.)

**Fig. 2:**
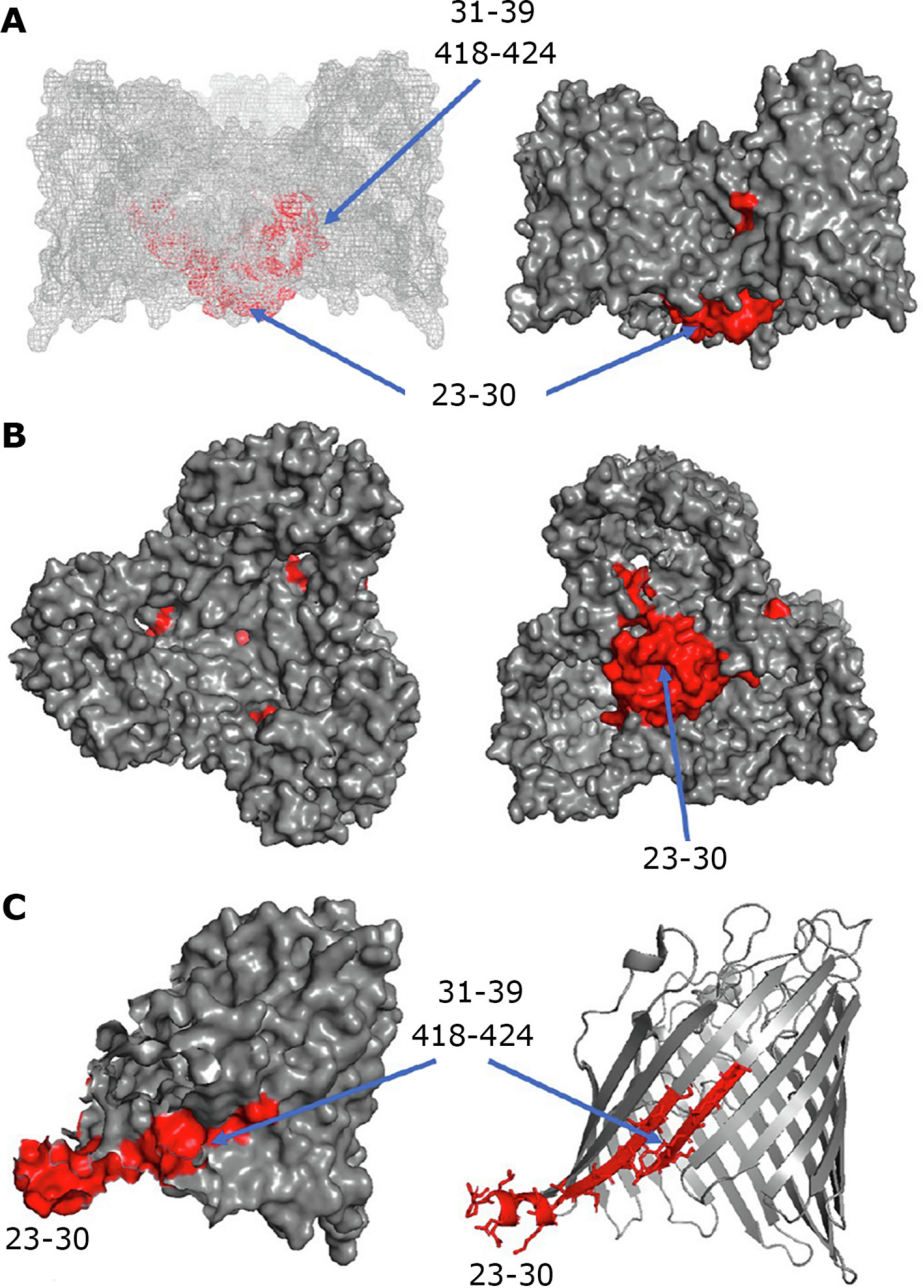
Regions that exhibit increased labelling upon QPLEX binding. The three regions (23–30, 31–39 and 418–424) with increased labelling (p ≤ 0.05) after addition of QPLEX are shown in red. **A.** Transmembrane side view of the MOMP trimer in mesh and space filling representations. **B.** Top view from the extracellular side (left) and bottom view from the intracellular side (right) in space filling representations **C.** Side view of a monomer in space filling and ribbon representations. The three regions (23–30, 31–39 and 418–419) are labelled and their locations are indicated by blue arrows. They form a continuum from the intracellular face moving through the porin towards the extracellular face. (For interpretation of the references to colour in this figure legend, the reader is referred to the web version of this article.)

Having optimised experimental conditions, carbene labelling of MOMP was assessed, in the presence and absence of 10 and 40 μM QPLEX, using trypsin. The presence of QPLEX caused significantly more labelling at peptides; 23–30, 31–39 and 418–424. Fractional modification of carbene labelling of peptide 23–30 increased from 0.2 to 0.65, peptide 31–39 from 0.03 to 0.07 and peptide 418 to 424 from 0.04 to 0.07 (Fig. 1). These significant changes in fractional modification (p ≤ 0.05) suggest that QPLEX binds to MOMP and induces conformational changes that expose these three regions to increased carbene labelling. Interestingly, no direct masking of carbene labelling due to binding of the QPLEX ligand was observed. This may result from the ligand binding site being small in area, or from ligand interactions with individual residues distributed across different tryptic peptides, which would dilute masking effects. It is also possible that the unmasking of residues, induced by conformational changes in MOMP, overrides any potential masking by QPLEX itself. The three unmasked regions of the protein were subsequently mapped on a structural model of the NCTC11168 MOMP (constructed from the crystal structure of MOMP 85H pdb 5LDT which is effectively the same protein with 89 % identity, 91 % similarity [14]), revealing a continuous extensive pathway running from the intracellular face (23–30) through the porin channel towards the extracellular face successively via the 31–39 and 418–424 regions (Fig. 2).

MOMP has four extracellular loops L1, L3, L4, and L6 which fold inside of the barrel, with L3, L4, and L6 at the constriction zone [14]. It is likely that the initial interaction of QPLEX is with some of the adjacent extracellular loops causing conformational changes that expose the 23–30, 31–39 and 418–424 regions. Similar extracellular loops of the homologous *Escherichia coli* OmpA protein mediate binding of antigens to immunoglobulins, toxins to protein receptors, metal ions to proteins, DNA to DNA-binding proteins and protein substrates to serine proteases [23]. Furthermore, loops L5 and L7 are important for membrane physiology in *Providencia stuartii* [24] while structural modification of the major porin at L3 can lead to antibiotic resistant strains, in *Enterobacteriaceae* [25]. An antibiotic resistant hypervirulent clone of *C. jejuni* was reported in the USA which caused significant problems in sheep farming by inducing abortions in pregnant sheep [26]. Allelic variations in L4 were identified as critical for the hypervirulence of this clone, indicating the important role of MOMP in Campylobacteriosis [27].

Residues 23–30 lie on the underside (the intracellular region) of MOMP on the *N-*terminus at the periplasmic face which has the β1 strand next to the *N-*terminal α1-helix that points away from the barrel wall. This region within the bacterial plasma membrane would not be accessible to small compounds or drugs from external sources, as it lies on the intracellular side of the protein in the periplasm. Regions 31–39 and 418–424 are located within the porin channel, with the former being closer to the intracellular face and the latter closer to the extracellular face, forming a continuum. Based upon our footprinting data, we hypothesize that QPLEX initially binds to the extracellular loops and induces conformational changes in the 31–39 and 418–424 regions, as it translocates through the porin channel (Fig. 2A and B) and finally emerging at the intracellular side close to the 23–30 region. The latter is next to a highly hydrophilic region (Fig. S3 and Fig. 2C) where QPLEX could emerge from and released into the periplasmic space.

### 2.3 QPLEX docking to the extracellular loops of MOMP

**Fig. 3:**
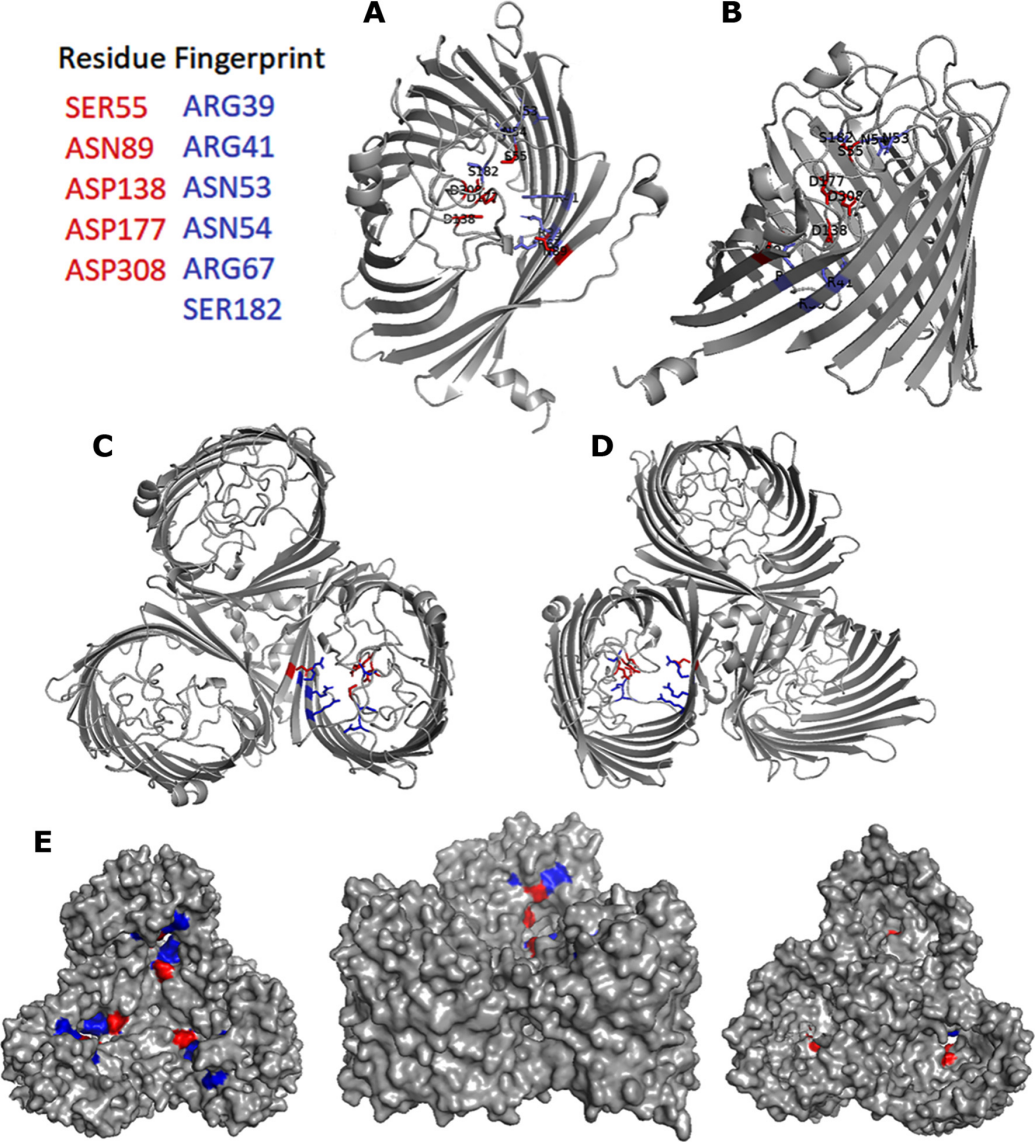
The *in silico* fingerprint of residues in the extracellular loop of MOMP that interact with QPLEX mapped onto the structural model of MOMP NCTC11168. In blue are amino acid residues that are hydrogen bond donors whilst in red are hydrogen bond acceptors from QPLEX. **A.** Extracellular view of the MOMP monomer in ribbon. **B.** Intermembrane side view of the MOMP monomer **C.** Extracellular view of the MOMP trimer in ribbon. **D.** Intracellular view of the MOMP trimer in ribbon. **E.** From left to right: extracellular view, intermembrane side view and intracellular view of the MOMP trimer. (For interpretation of the references to colour in this figure legend, the reader is referred to the web version of this article.)

To test our hypothesis the potential interaction of QPLEX with MOMP was studied further with docking studies, using FRED RECEPTOR 3.5.0.4 software (OpenEye Scientific Software, Inc., Santa Fe, NM, USA; www.eyesopen.com) and selecting Chemgauss4 functions for steric, clash, protein desolvation, ligand desolvation scores, as well as H-bonding interactions between ligand and protein. The Chemgauss4 scoring function uses Gaussian smoothed potentials to measure the complementarity of ligand poses within the active site and recognizes hydrogen bonding interactions between ligand and protein, interactions due to shape and hydrogen bonding interactions with the solvent. The more negative the magnitude of the Chemgauss-4 score, the tighter the ligand binding to the receptor through non-covalent interactions [28]. The MOMP 85H crystal structure (pdb 5LDT) was used for docking since it provides more geometric information than the MOMP NCTC11168 homology model and at 89 % sequence identity and 91 % similarity there will be limited loss of information. A comparison of the quality of docking using structural models and structural templates revealed that crystal structure templates gave better data compared to modelled structures [29]. QPLEX was docked on the extracellular loop region of MOMP and the top ranked pose was selected (Figs. S4-S6) revealing residues with which QPLEX is interacting (Fig. 3). QPLEX interacts with residues in the extracellular loop region of MOMP. Hydrogen bond donors to QPLEX in this region are Arg39, Arg41, Asn53, Asn54, Arg67 and Ser182 and hydrogen bond acceptors from QPLEX are Ser55, Asn89, Asp138, Asp199 and Asp308. The hydrogen bond interactions are the largest contributors to favourable binding in the extracellular loop region, contributing a score of −11.91 (Fig. S4).

### 2.4 QPLEX docking into the porin channel of MOMP

**Fig. 4:**
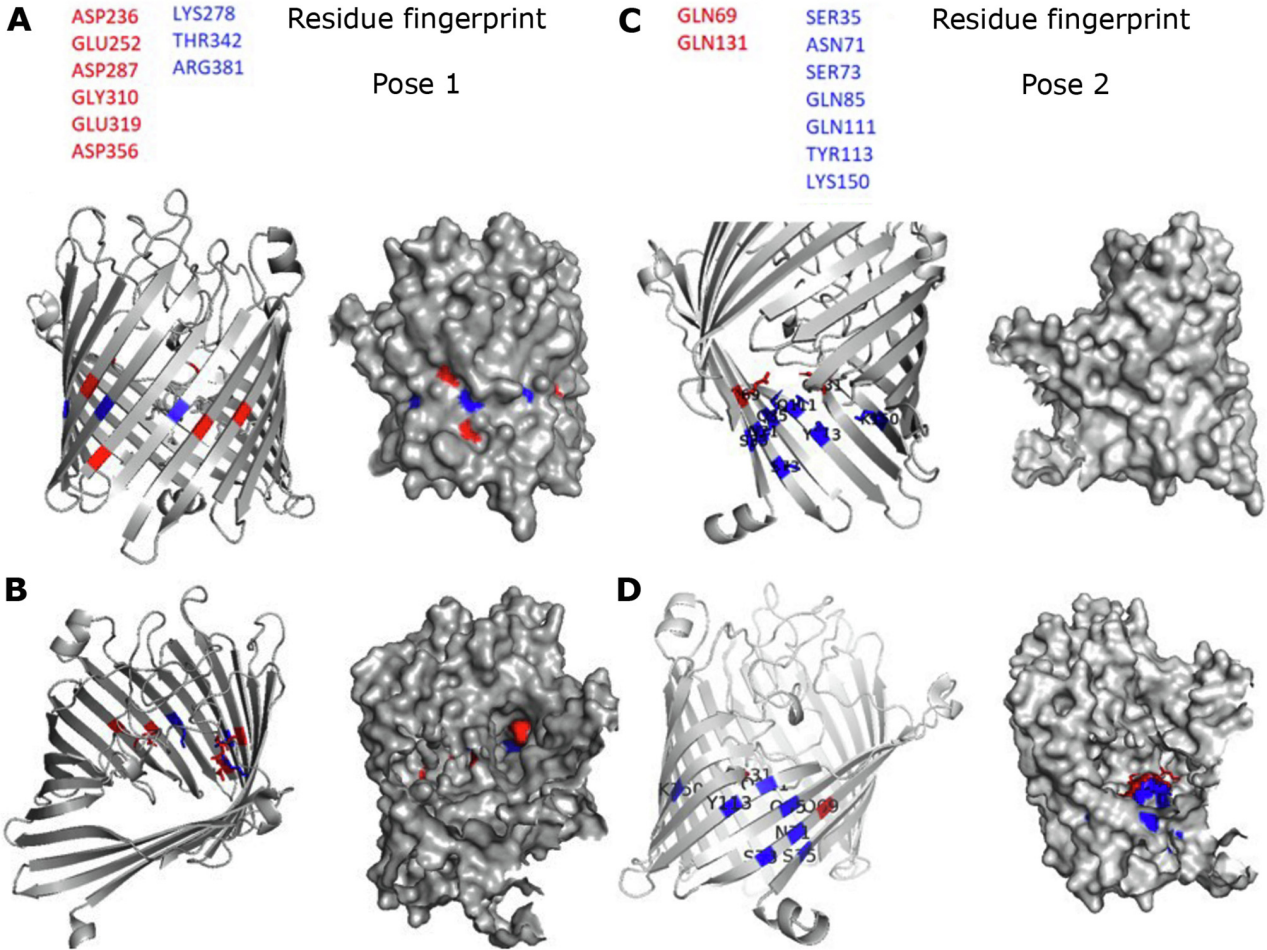
The *in silico* fingerprint of residues within the porin channel of MOMP in Pose 1 (Panels **A**, **B**) and Pose 2 (Panels **C**, **D**) that interact with QPLEX mapped on the homology model of MOMP 11168. In red are hydrogen bond acceptors from the QPLEX, in blue hydrogen bond donors by MOMP. Ribbon representations and space filling of MOMP 11,168 monomer are shown from the intermembrane view (**A, C**) and the extracellular view (**B, D**) of the monomer. (For interpretation of the references to colour in this figure legend, the reader is referred to the web version of this article.)

Carbene footprinting also suggested QPLEX-induced conformational changes and passage through the channel of the porin to the intracellular side. To further address this issue, we performed docking studies by creating a box along the length of the porin channel excluding the extracellular and intracellular regions. The top two poses from triplicate experiments were selected (Figs. S7 and S8), revealing several residues along the channel of the porin that could potentially interact with QPLEX (Fig. 4) Interestingly, Pose 1 (Fig. 4A and B) is located closer to the extracellular surface of the porin channel whilst Pose 2 (Fig. 4C and D) is closer to the intracellular side. The overall FRED Chemgauss4 score shows that binding of QPLEX to the porin is favourable in these regions. The score is similar to that of docking QPLEX to the extracellular loop region, owing to the continuity of transient and weak hydrogen bonds that are formed as QPLEX travels down, through the channel of the porin. Both the shape and hydrogen bond scores between MOMP and QPLEX contribute significantly to the magnitude of the negative overall score. It would be consistent with our docking data to conclude that QPLEX moves through the channel of the porin, after initial binding to the extracellular loops, towards the intracellular face.

Pose 1 region is surrounded by negatively charged residues while Pose 2 region is surrounded by positively charged residues thus forming a dipole across the axis of the elliptical constriction zone of the porin, termed the transverse electric field. This transverse field has been shown to be important for the translocation of polar molecules from the environment into gram-negative bacterial cells. It is an important characteristic involved in drug design where zwitterionic compounds, such as penicillin and ciprofloxacin, are able to use the polarity of the porin to pass through the constriction zone [30]. When we align and blast the amino acid fingerprint onto our homology model of MOMP NCTC11168, the QPLEX interacting residues in the top half of the porin are Asp236, Glu252, Asp287, Gly310, Glu319, Asp356, Lys278, Thr342 and Arg381 (Pose 1 As QPLEX moves further down into the channel towards the intracellular side, it interacts with Gln69, Gln131, Ser35, Asn71, Ser73, Gln85, Gln111, Tyr113 and Lys150 (Pose 2 It is likely that as QPLEX travels down the channel and interacting with these residues it induces conformational changes that render the 31–39 and 418–424 regions more susceptible to carbene footprinting.

### 2.5 QPLEX docking to the intracellular side of MOMP

In the presence of QPLEX, there was an increase in carbene labelling at the intracellular 23–30 region of the protein that faces the periplasmic space. We performed additional QPLEX docking to investigate whether QPLEX could be bound to this region of the protein. The top ranked pose was selected (Fig. S9) and the data indicated that binding of QPLEX to this region is unfavourable, with a FRED Chemgauss score of 1.5. A large part of this score was due to lack of steric interactions in this region which meant there was less instance of hydrogen bonding to be able to overcome the MOMP and QPLEX desolvation score due to a highly hydrophilic environment in the periplasm. This suggests that the translocation of QPLEX through MOMP induces conformational changes at the intracellular face of MOMP rather than binding to this region in a similar manner it binds to the extracellular loops. Furthermore, these data suggest that QPLEX travels through MOMP and is readily released within the periplasmic region rather than remaining bound to the intracellular face of MOMP.

## 3 Discussion

Antibiotic resistance is a global problem bringing enormous challenges to human health. The wide use of antibiotics globally in preventative feeding regimes during animal farming has contributed significantly to the emergence of antibiotic resistance. *C. jejuni* infections in commercial broiler farming is a serious problem with infected chickens at the supermarket shelves being the main source of Campylobacteriosis infections in humans. *C. jejuni* antibiotic resistance in humans is intimately linked to this transmission route. Studies have shown that the number of macrolide-resistant and fluoroquinolone-resistant Campylobacter spp. isolates from humans is influenced by factors including veterinary use of macrolides and fluoroquinolones [31]. Fluoroquinolones were widely used in human medicine in the Netherlands and the US before they were approved for animal use, but prior to animal use there was no emergence of quinolone resistance in Campylobacter spp. in humans. Although human macrolide and fluoroquinolone clinical use contributes to increased antibiotic resistance, their relative contribution to increased resistance compared to the use of these agents in animal husbandry appears to be small [32].

While global efforts to control transmission of enteric pathogens have been effective at reducing the incidence of major foodborne pathogens, the prevalence of Campylobacter infections has continued to increase across most developed nations. The indiscriminate preventative use of antibiotics is rendering infection control increasingly inefficient, and the rise of antibiotic resistance signals a need to develop other types of intervention. Ferric chelates show great promise as feed additives in animal farming [17], [18], [19]. They appear to have potent anti-biofilm activity and significantly reduce Campylobacter loads in commercial broilers. Because they do not exhibit bactericidal or bacteriostatic activities, they are unlikely to promote the development of resistance. Their precise mechanism of action is not known but it was suggested that they may act by preventing MOMP-mediated attachment of bacteria in the chicken gut epithelia. Their structures resemble bacterial siderophores which are transported through outer membrane porins (OMPs) into the periplasmic space and then through the inner cell membrane into the cell via ABC transporter systems. The TonB-ExbB-ExbD energy transduction system provides energy to transport siderophores across the outer membrane in Gram negative bacteria, with TonB transducing the energy from the proton motive force of the inner membrane to the outer membrane receptors to allow for siderophore import [33], [34], [35]. Siderophore transport in *C. jejuni* also appears to be TonB dependent and interestingly TonB1 and TonB3 appear to be essential for commensal broiler colonization while TonB2 is dispensable [36].

**Fig. 5:**
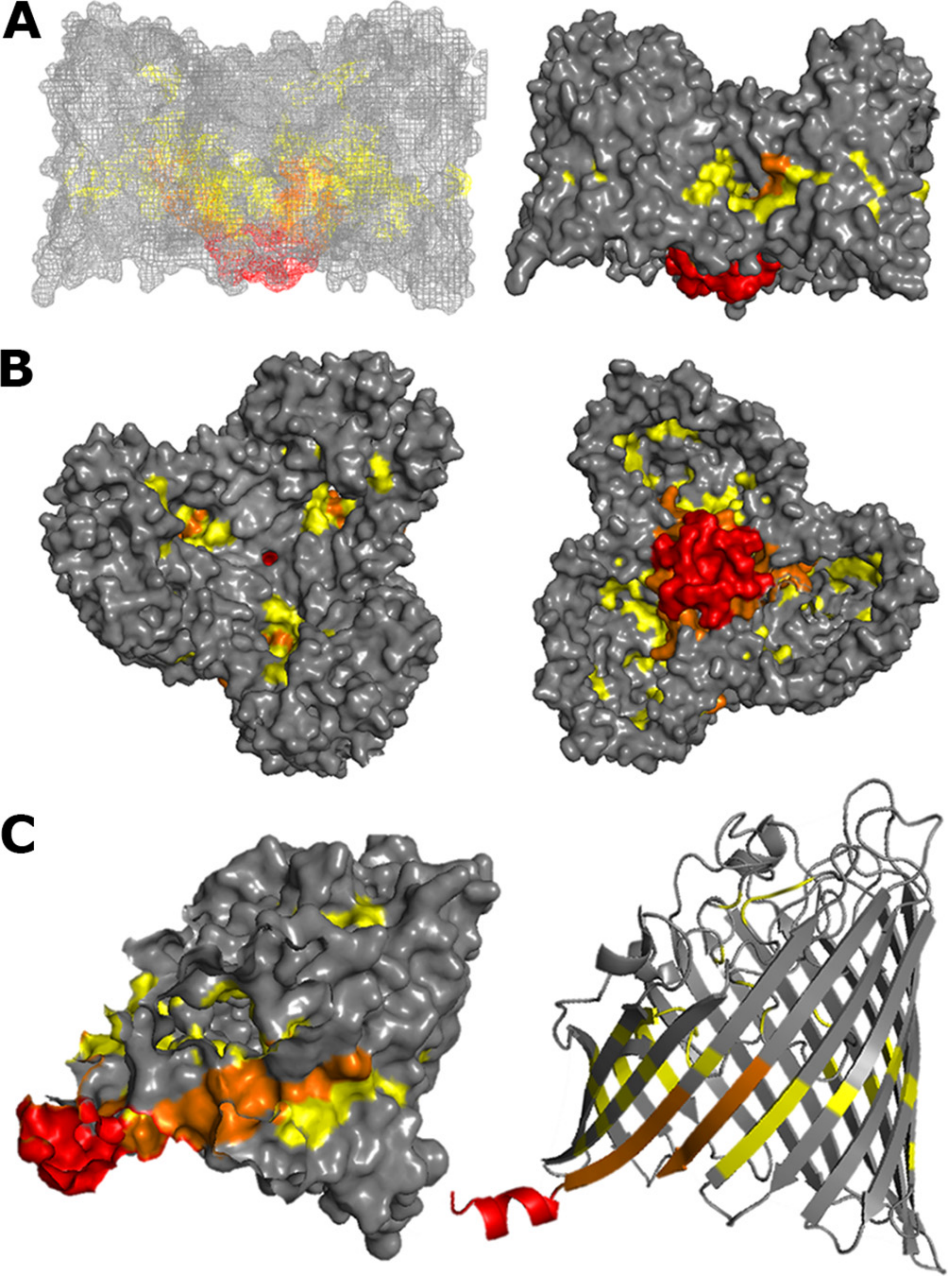
Combined mapping of all the regions identified by carbene footprinting and *in silico* docking: A structural model for QPLEX binding to MOMP and translocation through the porin channel.The three regions (23–30, 31–39 and 418–424) with increased labelling (p ≤ 0.05) after addition of QPLEX are shown in red in the same orientations as in Fig. 2. Regions identified via *in silico* docking are shown in yellow and overlapping regions identified by both carbene footprinting and *in silico* docking are shown in orange. **A.** Transmembrane side view of the MOMP trimer in mesh and space filling representations. **B.** Top view from the extracellular side (left) and bottom view from the intracellular side (right) in space filling representations. **C.** Side view of a monomer in space filling and ribbon representations. (For interpretation of the references to colour in this figure legend, the reader is referred to the web version of this article.)

Here, we show that the ferric chelate QPLEX binds to MOMP, a major virulence factor of *C. jejuni*, which exhibits selectivity and functions as a control channel for the bidirectional transport of nutrients and other molecules through the outer membrane into the periplasm [37]. Carbene footprinting revealed that QPLEX binding to MOMP induces increased fractional labelling to three regions, 23–30, 31–39 and 418–424 (Fig. 2). These regions form a continuum running from the intracellular face of MOMP (region 23–30) into the porin channel towards the extracellular face successively through regions 31–39 and 418–424. *In silico* docking guided by our carbene footprinting revealed QPLEX interactions with the extracellular loops and with a transverse electric field, comprising of the negatively charged Pose 1 region in the upper part of the porin channel and the positively charged Pose 2 region in the lower part of the channel (Fig. 3, Fig. 4). Interestingly, docking studies of QPLEX at the intracellular face of MOMP indicates that MOMP does not stably bind to this region which is consistent with its release into the periplasm (Fig. S9). To increase selectivity of small molecules that pass through the porin, in MOMP it was shown that there is a Ca^2+^ ion in the constriction zone that can work to reverse the transverse field [14]. It is not known whether such an ion exists in our MOMP 11168, thus Ca^2+^ was not included in our docking studies. However, if an ion were to be present, it would not have a large effect on the entrance of the QPLEX compound into the constriction zone due the non-ionic nature of QPLEX, making it a powerful tool for entering the porin. Considering our combined carbene footprinting, *in silico* docking data and mapping all the interactions on the structure of MOMP, suggests that QPLEX interacts first with the extracellular loops of MOMP inducing considerable conformational changes that allow it to translocate through the porin channel and enter the periplasmic space via the 23–30 intracellular region. Mapping of all regions identified by carbene footprinting and *in silico* docking reveal the detailed binding of QPLEX to MOMP and subsequent translocation path through the porin channel (Fig. 5).

It is still unknown how QPLEX then enters in the cell through the inner membrane and whether it induces downstream pleiotropic physiological effects in *C. jejuni* which might alter its virulence. We propose that ferric chelates that are structurally equivalent to QPLEX and TYPLEX, enter *C. jejuni* via the same mechanism. Binding of these ferric chelates to MOMP may also antagonise the interaction of MOMP with BgAgs for attachment to the gut epithelia of broilers. This offers an explanation for the 2–3 log_10_ reduction of *C. jejuni* loads observed when TYPLEX was used as feed additive in broiler farming [17], [18], [19]. Future studies should be directed at mapping the interactions of BgAgs with MOMP, revealing the mechanism of internalisation of these ferric complexes into the cytoplasm through the inner membrane and investigating whether they induce downstream physiological changes in *C. jejuni*.

## 4 Methods

### 4.1 Purification of native MOMP

Native MOMP was extracted and purified directly from *C. jejuni* NCTC11168 cells and verified by Western blots (Fig. S10). *C. jejuni* NCTC11168 was grown from a glycerol stock on a Campylobacter selective blood free agar (CCDA) plate (Oxoid) under microaerophilic conditions for 48 h at 42° C. To ensure sufficient biomass for MOMP purification, growth on the single plate was subsequently amplified to 15 further plates and then to 450 plates in total.

Bacteria from each plate were harvested and rinsed with 5 ml of 10 mM Tris, 1 mM EDTA (pH 7.2) for 15 min at room temperature with gentle agitation. The following steps were carried out at 4 °C. After centrifugation at 10,000× g for 30 min, cell pellets were washed in 200 mM glycine-HCl (pH 2.2) to eliminate proteases and associated outer membrane proteins, and agitated for 15 min, following by another round of centrifugation at 10,000× g. The bacterial pellet was washed twice successively in 100 mM Tris (pH 7.2) to titrate excess HCl. Then the supernatant was discarded, and cells were suspended in 10 mM Tris HCl (pH 7.2) and sonicated in the same buffer. The sonication was carried out at 30 kHz with 10 rounds of 30 s alternatively on and off on ice. After sonication, cell fragments were removed by centrifugation (6000× g for 30 min) and the total membrane fraction (the supernatant) was recovered by ultracentrifugation at 100,000× g for 1 h at 4 °C. The pellet was homogenized in 10 mM Tris (pH 7.4) and 0.1 % (w/v) of SDS (Sigma) to solubilize the inner membrane and left rocking for 30 min. The outer membrane was recovered by ultracentrifugation at 100,000 g for 1 h. The supernatant containing the inner membrane protein fraction was discarded, and the pellet was homogenized with 20 mM sodium phosphate buffer (pH 7.4) and 1 % (v/v) of poly(ethylene glycol) octyl ether (Octyl-POE) (Bachem AG) and left rocking at 4 °C for 30 min. Hereafter, Octyl-POE was used as detergent to keep MOMP in solution. Ultracentrifugation at 100,000 g for 1 h led to the recovery of solubilised MOMP in association with POE micelles. MOMP was purified through a MonoQ HR anion exchange column (GE Healthcare), equilibrated with 5 Column Volumes (CV) of buffer A (30 mM Na_2_HPO_4_, 10 mM NaCl, and 0.6 % (v/v) Octyl-POE The bound proteins were eluted stepwise with 5, 12, 20, 70 and 100 % of buffer A supplemented with 1 M NaCl. Each fraction was examined with SDS PAGE and Western blot with specific rabbit anti-MOMP antibody. Eluted fractions containing MOMP were collected, concentrated to 5 ml and injected onto a Superdex S200 16/60 GL (GE Healthcare) gel filtration column equilibrated with 2 CV of 10 mM Tris (pH 8.0), 150 mM NaCl, and 0.45 % (w/v) C8E4. Fractions containing MOMP were combined and concentrated with a spin concentrator. A nanodrop was used to measure protein concentration. A typical yield per 150 plates was 6.8 mg of MOMP which was verified by Western blots, as described below.

### 4.2 Western blots

Western blots were carried out with anti-MOMP-specific rabbit antibody. Transfer from polyacrylamide gels to nitrocellulose membranes was carried out either by the semi-dry or wet transfer methods. After transfer of proteins to the nitrocellulose membrane, the membrane was blocked for 1 h at room temperature, using blocking buffer (5 % w/v skimmed milk powder in 20 ml PBS Tween20 The membrane was incubated overnight at 4 °C in 1:2,000 dilution of primary anti-MOMP rabbit antibody in blocking buffer. The membrane was washed in three washes of PBST, for 5 min each and then incubated with 1:3,000 dilution of conjugated secondary antibody (anti rabbit IgG alkaline phosphatase conjugated antibody) in blocking buffer at room temperature for 1 h. The membranes were washed in three washes of PBST, for 5 min each, and bands were visualised by adding alkaline phosphatase substrate (1-Step NBT/BCIP The gels were scanned in the BioRad Gel imager system using upper white light (Fig. S10).

### 4.3 Synthesis of ferric quinate (QPLEX)

QPLEX was synthesized by mixing quinic acid and ferric chloride in a 3:1 M ratio, as described elsewhere [38], 0.50 g, 1.8 mmol of FeCl_3_·6H_2_O was placed in a flask and dissolved in 3 ml of H_2_O. To that solution, D-(-)-quinic acid (1.1 g, 5.5 mmol) was added slowly, under continuous stirring. KOH was added slowly to adjust the pH to ∼3. The resulting reaction solution was stirred for 1 h at room temperature. Ethanol was then added, and the reaction mixture was placed at 4 °C. A few days later, yellowish cubic QPLEX crystals appeared at the bottom of the flask. The QPLEX crystals were isolated by filtration and dried under vacuum. HPLC (three serial columns C5-C8-C8, 150 x 46 mm, in 100 mM aqueous phosphoric acid) was used to check the purity of the compound (Fig. S11).

### 4.4 Synthesis of the sodium-TDBA carbene footprinting probe

A solution of sodium hydroxide (16 mg, 0.39 mmol) in H_2_O (1 ml) was added to 4-(3-(trifluoromethyl)–3H-diazirin-3-yl) benzoic acid (TDBA) (100 mg, 0.43 mmol) in an Eppendorf tube and thoroughly mixed to form a sodium salt of TDBA (Fig. S12).

### 4.5 Carbene footprinting

For carbene labelling in the absence of the QPLEX ligand, an aqueous solution of NCTC11168 native MOMP (10 μM) in 1 % (v/v) octyl-POE and PBS was mixed with an equal volume of a solution of the aryl diazirine probe (100 mM) in 20 mM Tris, 150 mM NaCl, pH 7.4, to give a final probe concentration of 50 mM and protein concentration of 5 µM. For carbene labelling in the presence of the QPLEX ligand, an aqueous solution of NCTC11168 MOMP (10 μM) in 1 % (v/v) octyl-POE in PBS and 10 µM QPLEX in PBS was mixed with an equal volume of a solution of aryldiazirine (100 mM) in 20 mM Tris, 150 mM NaCl, pH 7.4, to give a final probe concentration of 50 mM, protein concentration of 5 µM and QPLEX concentration of 5 µM in 5 µl solution.

In order to carbene label the MOMP protein, 5 μl aliquots of the above samples were placed in tapered X100 crystal clear vials (ThermoFisher Scientific, Paisley, UK) and flash-frozen with liquid nitrogen. Each sample was irradiated for 16 s using a Spectra Physics Explorer 349 laser (an actively Q-switched Nd:YLF laser operating at 349 nm, with a repetition frequency of 1000 Hz, and a pulse energy of 125 μJ, Newport, Didcot, UK The laser beam was directed into the open top of the sample vial using a small 45° mirror. The same laser conditions were used to irradiate a solution of MOMP in presence and absence of 1 % (v/v) OG using 5 mM, 10 mM, 25 mM or 50 mM concentrations of diazirine. All labelling experiments were repeated in triplicate and the labelling process is explained further in Fig. S13.

All carbene labelled proteins were purified and visualized by SDS PAGE. Protein bands were excised from the gel and destained thoroughly by ammonium bicarbonate followed by dehydration by acetonitrile until 1 mm^3^ dehydrated white pieces of gel saturated with protein remained. These were then incubated overnight at 37 °C for 16 h by covering them with a solution of the enzyme in 50 mM ammonium bicarbonate and final concentration of 20 µg/ml of trypsin or 2 µg/ml chymotrypsin. After overnight incubation, the reaction was stopped with 1 % (v/v) formic acid and the samples were spun down at 3,000xg for 3 min to separate gel pieces from the solution. The supernatant was decanted into new LC vials for analysis by nanoHPLC-MS.

### 4.6 LC-MS and LC-MS/MS analysis

Protein digests were analysed in a Dionex U3000 nanoLC coupled to a ThermoFisher LTQ FT Ultra mass spectrometer. Samples (2 μl) were injected in load-trapping mode. Peptides were eluted using a 30 min linear gradient from the initial mobile phase A (5 % v/v acetonitrile, v/v 0.1 % formic acid) to 55 % mobile phase B (95 % v/v acetonitrile, 0.1 % v/v formic acid) followed by 5 min at 90 % B and 15 min column (Analytical: Pepmap 300 C18, 0.075×150 mm, nano-column; Trapping: Pepmap 300 C18, 0.3×10 mm, trap cartridge) re-equilibration. The LTQ FT Ultra mass spectrometer was equipped with a nanoelectrospray (nESI) source through which a 1.7 kV voltage was applied to the Picotip emitter. The inlet capillary of the mass spectrometer was held at 275 °C with a tube lens value of 145 V [21]. For labelled peptides and in order to quantify the modification of these peptides by carbine footprinting, samples were separated and data acquired in full scan mode followed by data analysis with PepFoot software [22]. Representative data, visualized with PepFoot, are shown in Fig. S14. For non-labelled protein, nanoHPLC-MS was operated in data directed analysis (DDA) mode, where a low energy survey was used to identify precursors of interest by intensity, charge state and isotope pattern. When precursors of interest were identified, MS/MS was performed on these peptides to obtain fragmentation data. Precursor peptides that had no MS/MS data were targeted in another round of experiments through targeted DDA. The data were submitted to X!Tandem search engine via SearchGUI and searched against a custom database including the wtMOMP and MOMP^T/G268^ sequence.

### 4.7 *In silico* studies

Protein ligand docking was performed via FRED RECEPTOR 3.5.0.4 software (OpenEye Scientific Software, Inc., Santa Fe, NM, USA A structural model of the NCTC11168 MOMP was created using the SwissProt Modeller and the crystal structure of the highly homologous (89 % identity, 91 % similarity) MOMP 85H (PDB 5LDT) as template. For docking in OpenEye FRED, the ChemDraw files of the ligand molecules, QPLEX was converted to a conformationally expanded 3D format such as SDF, MOL2 or PDB. This was performed by copying their ChemDraw structures as SMILES and converting to 3D structures using Openbabel file conversion software. As OpenEye FRED requires a single receptor to dock ligands, the MOMP was processed as a receptor in MakeReceptor as described in Fig. S15.

Docking of QPLEX was performed using FRED in OpenEye’s command line utility and the MOMP 85H and QPLEX files prepared previously. High resolution of the exhaustive search was used to dock the ligand where translational and rotational step sizes were set to 1 Å. Docked results were visualised by VIDA 4.4.0.4 where the top ten resultant ligand poses and translations were placed in the active site of MOMP.

**Data accessibility**

Mass spectrometry raw data have been deposited to the ProteomeXchange Consortium (https://proteomecentral.proteomexchange.org) via the PRIDE partner repository with the dataset identifier PXD035978 (http://www.ebi.ac.uk/pride/archive/projects/PXD035978).

## Declaration of Competing Interest

The authors declare that they have no known competing financial interests or personal relationships that could have appeared to influence the work reported in this paper.
