# Supplementary material for: Ferric quinate (QPLEX) interacts with the major outer membrane protein (MOMP) of Campylobacter jejuni and enters through the porin channel into the periplasmic space
Source: Comput Struct Biotechnol J. 2022 Sep 24;20:5355–63. doi: 10.1016/j.csbj.2022.09.032 (PMC9522878; doi:10.1016/j.csbj.2022.09.032)
Supplement: Supplementary data 1 [file mmc1.docx]

**SUPPLEMENTARY INFORMATION**

**Ferric Quinate (QPLEX) interacts with the Major Outer Membrane Protein (MOMP) of *Campylobacter jejuni* and enters through the porin channel into the periplasmic space**

Jennifer C. Okoye^1^, Jeddidiah Bellamy-Carter^2^, Neil J. Oldham^2^, Neil J. Oldfield^3^, Jafar Mahdavi^1^, Panos Soultanas^1,^*

^1^School of Chemistry

Biodiscovery Institute

University of Nottingham

University Park

Nottingham

NG7 2RD

UK

^2^School of Chemistry

University of Nottingham

University Park

Nottingham

NG7 2RD

UK

^3^School of Life Sciences

University of Nottingham

Queens Medical Centre

Derby Road

Nottingham

NG7 2UH

UK

*Corresponding author: panos.soultanas@nottingham.ac.uk


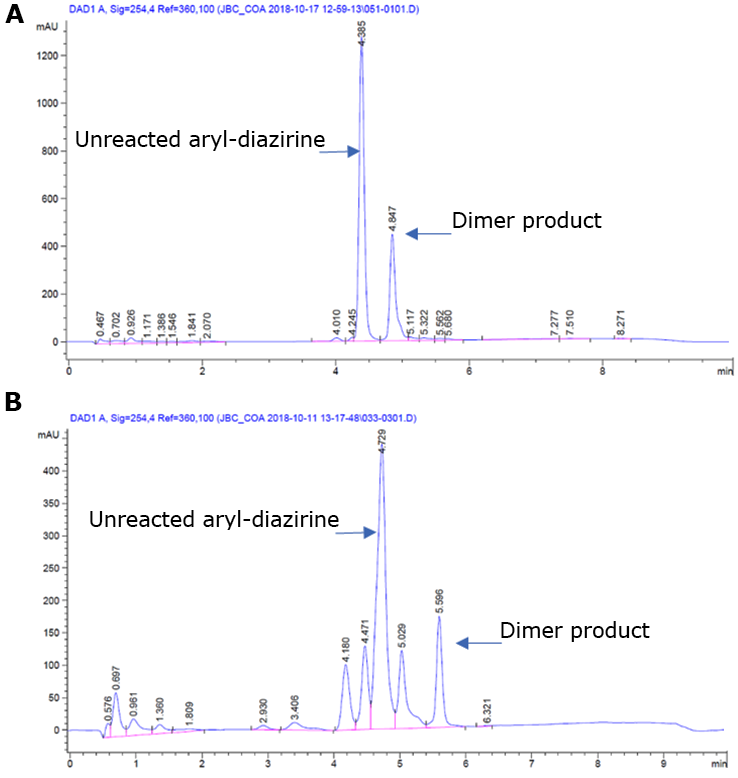


**Figure S1:** Labelling efficiency by aryl diazirine in the presence of non-ionic detergents. **A.** 50 mM 4-(3-Trifluoromethyl)-3H-diazirin-3-yl)benzoic acid (TDBA) was mixed with 0.45% (v/v) *O-*POE and then irradiated for 16 seconds before analysed by Liquid Chromatography (wavelengths were modified to observe probe). **B.** A similar experiment as described in panel A with 50 mM TDBA mixed with 0.45% (v/v) OG.


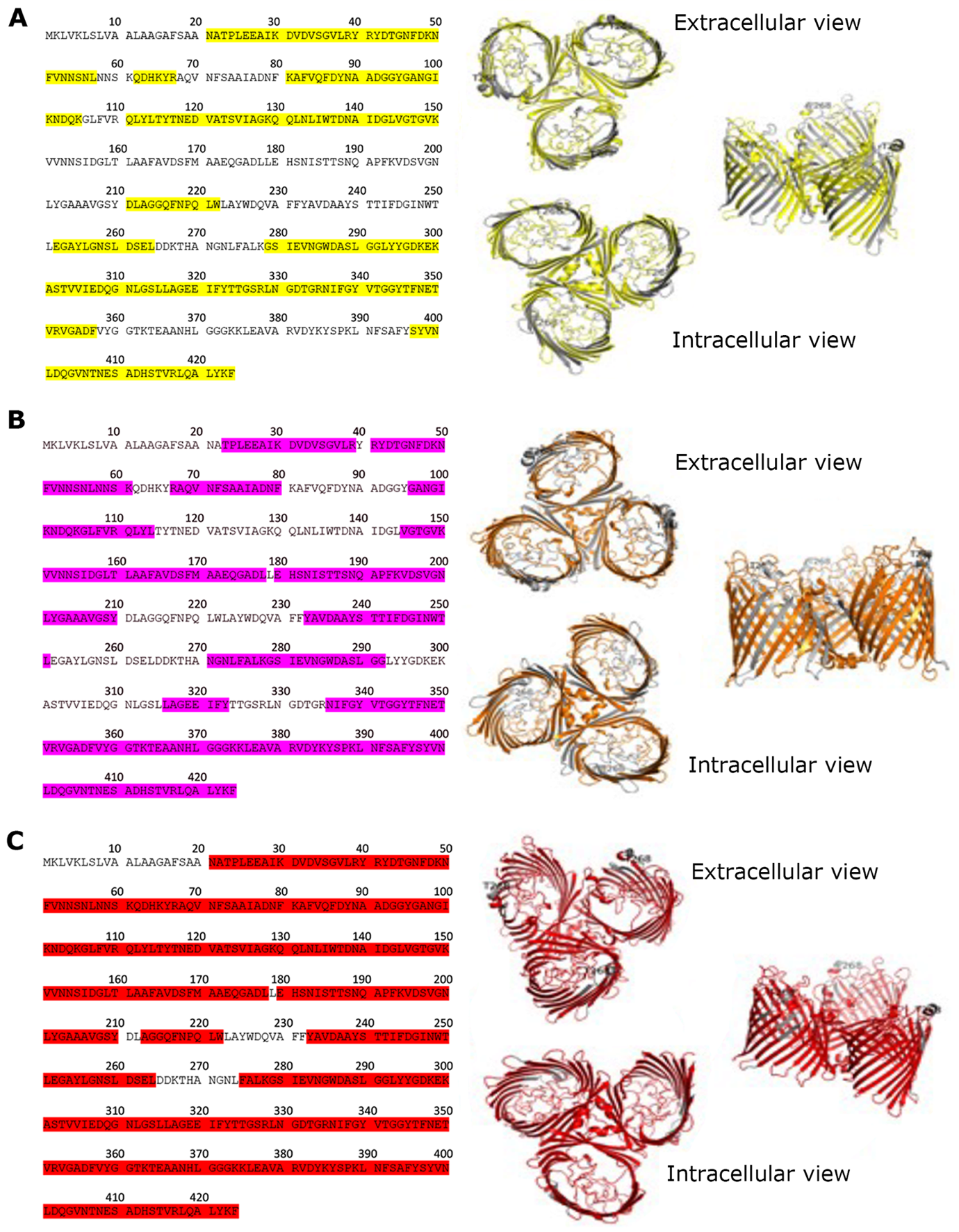


**Figure S2:** Optimisation of carbene labelling for MOMP.

**A.** Total peptide coverage from trypsin and chymotrypsin digestion of unlabelled peptides that were identified but not carbene labelled, highlighted in yellow and mapped with colour onto the model structure of MOMP NCTC11168. It is worth noting that the signal peptide of MOMP is predicted to be cleaved between Ala22 and Thr23 hence the lack of coverage at the N-terminus is expected **B.** Peptides from trypsin and chymotrypsin digestion that were found to be carbene labelled, highlighted in pink and mapped with colour onto the model structure of MOMP NCTC11168. **C.** All identified peptides labelled or unlabelled from both chymotrypsin and trypsin highlighted in red and mapped with colour onto the model structure of MOMP NCTC11168. The MOMP NCTC11168 structure was modelled onto the crystal structure of MOMP 85H (PDB 5LDT) (Ferrara et al., 2016) with threonine 268 involved in *O*-glycosylation labelled. The structures are shown from extracellular and intracellular views, as indicated, as well as from the intermembrane view with the extracellular and intracellular faces on the top and bottom, respectively.


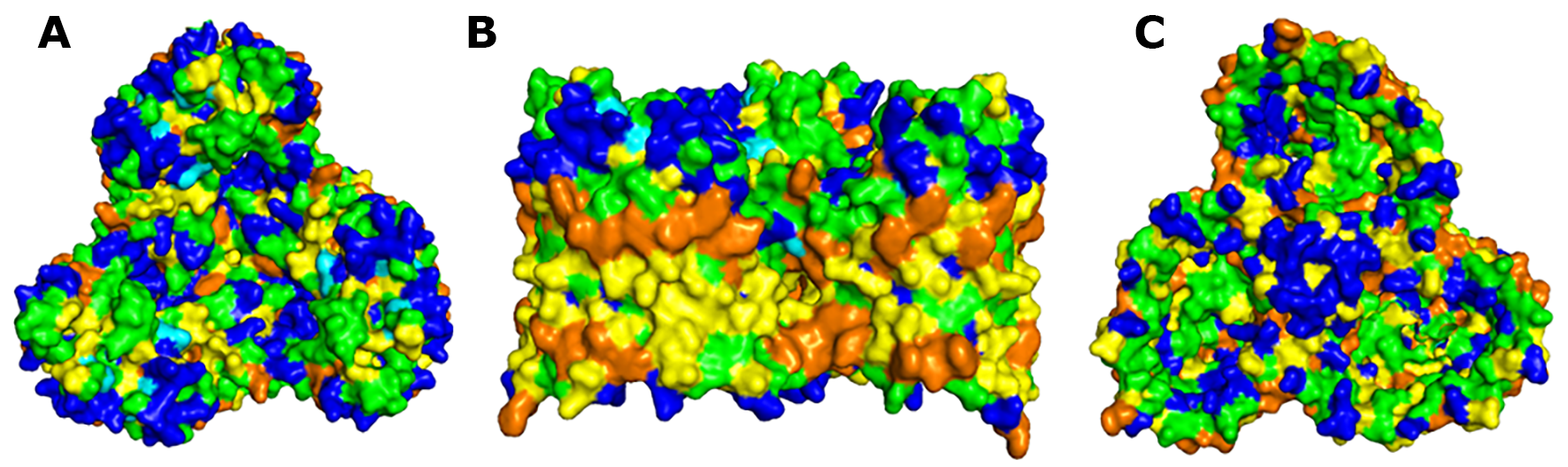


**Figure S3:** Hydrophilic regions on the surface of MOMP are shown as green and blue whilst hydrophobic regions shown as yellow and orange patches on the protein surface of MOMP. Colour code: Orange = Tyr+Phe+Trp, yellow = Leu+Ile+Val+Met+Pro+Ala+Cys, green = Asn+Gln+Ser+Thr+Gly, cyan = His, blue = Arg+Lys+Asp+Glu. Protein orientation: **A.** Extracellular view. **B.** Side view. **C.** Intracellular view.

**
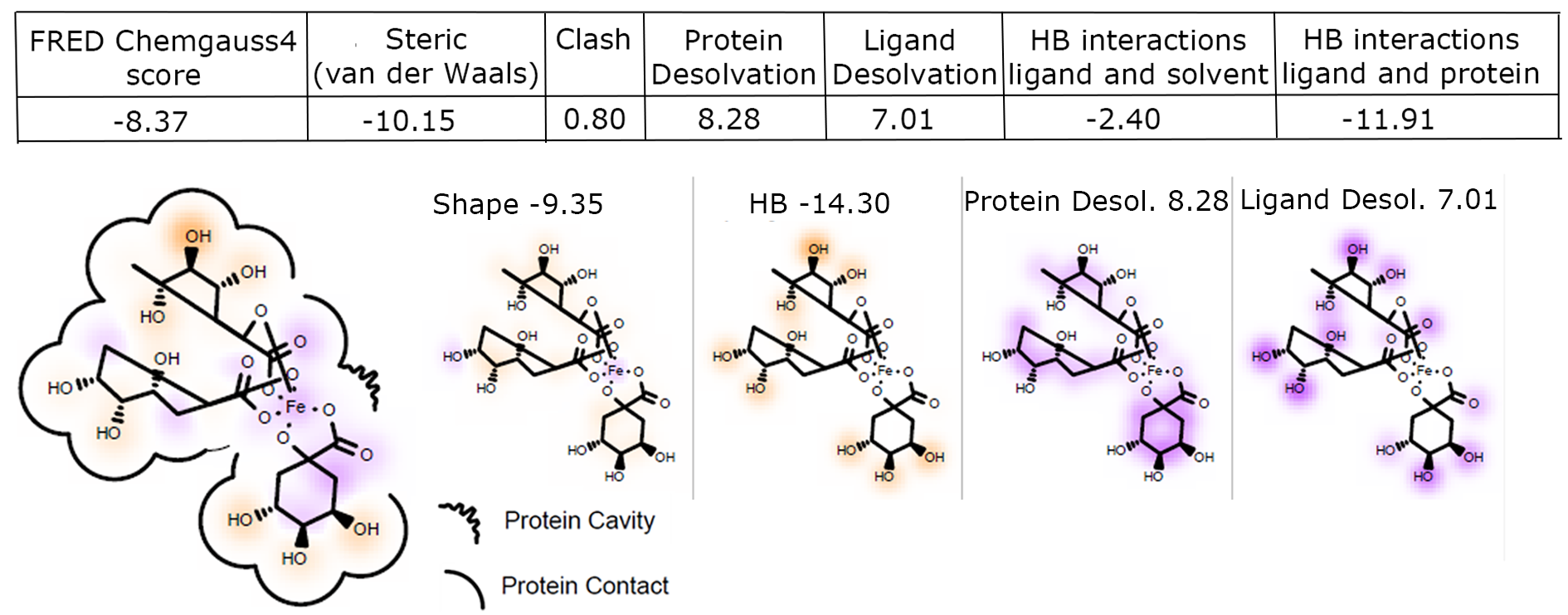
**

**Figure S4:** The report of the FRED Chemgauss4 score for the top pose when docking QPLEX on the extracellular loop region of MOMP is shown at the top. On the bottom a breakdown of the components that contribute to the overall FRED score (shape score, hydrogen score, protein desolvation score and ligand desolvation score) is shown. The shape score is a function of the steric (van der Waals) and clash score. The hydrogen bond schematic highlights the hydrogen bonds between QPLEX and MOMP or QPLEX and solvent. MOMP/QPLEX desolvation shows the penalty from the interaction of water in that pose/translation. The degree of interaction is represented by an orange to purple scale bar where orange indicates a stronger interaction and purple a weaker interaction.


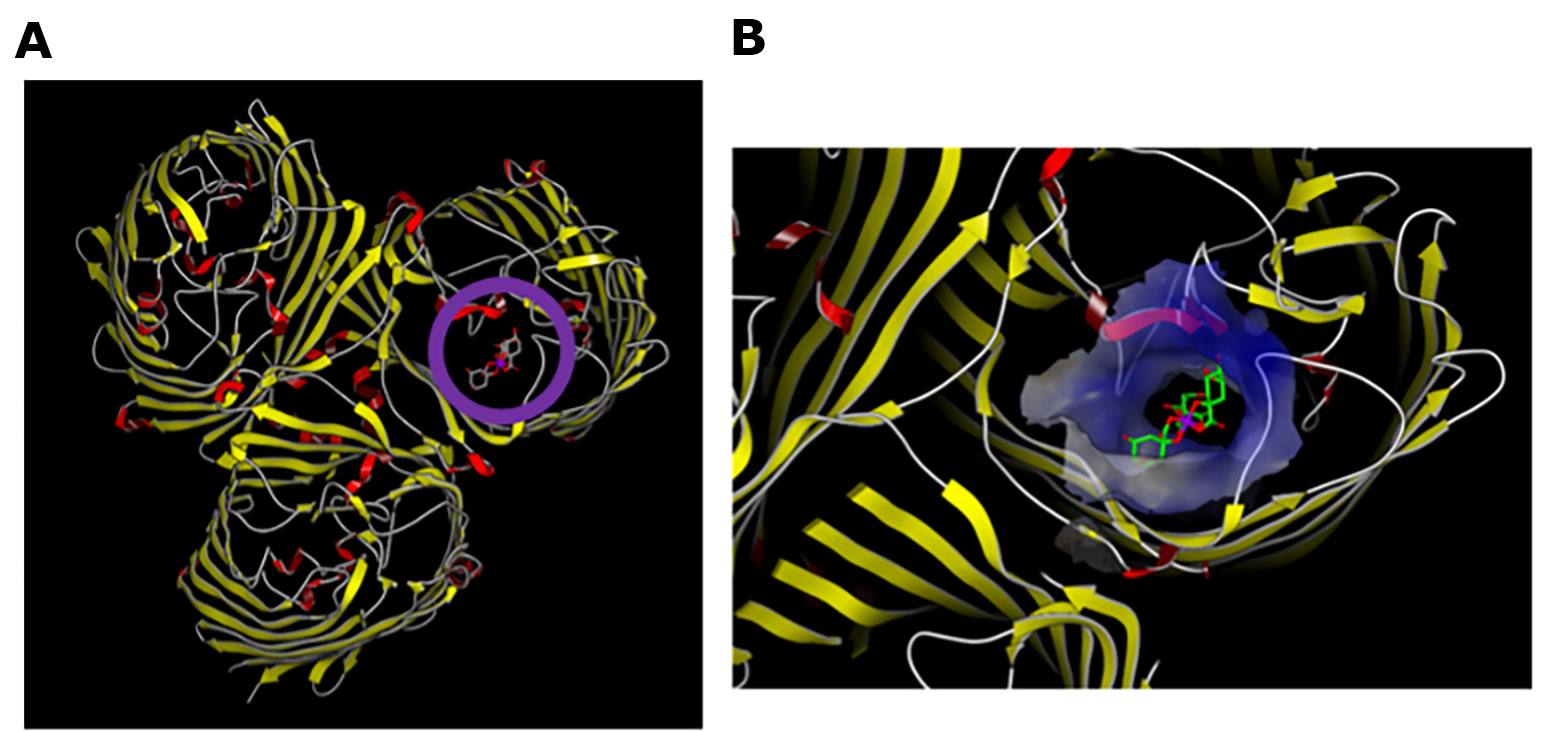


**Figure S5:** **A.** Docking result of the highest ranked pose of QPLEX on extracellular surface of MOMP. **B.** The secondary structure in this site is random loop and so the surface of the protein has been included for easier definition of the site.


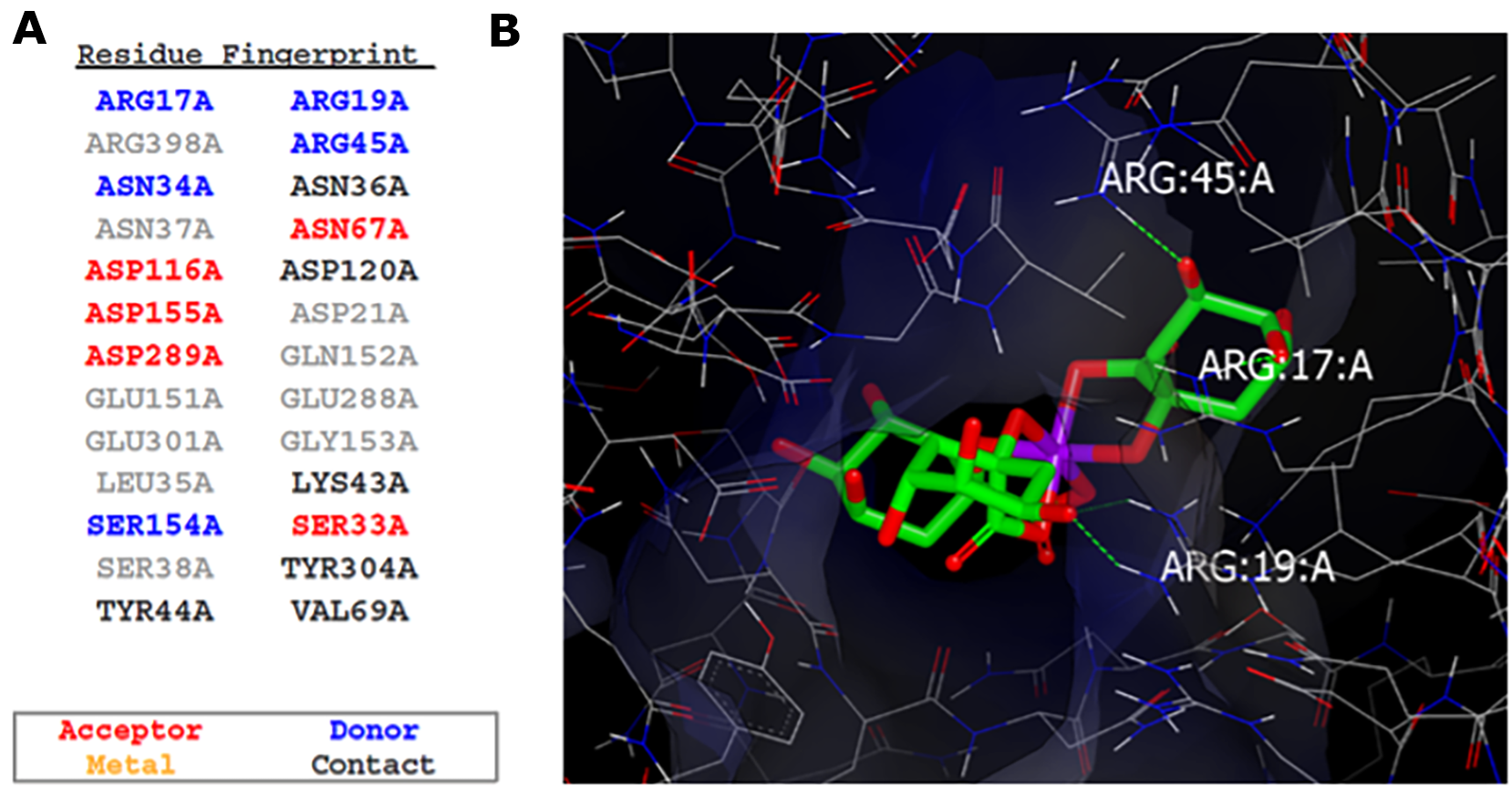


**Figure S6:** **A.** Residues in the MOMP extracellular site that QPLEX interacts with. Red denotes amino acid residues that accept hydrogen bonds from QPLEX, blue denotes hydrogen bonds that are donated by MOMP, black denotes residues on MOMP that QPLEX contacts and other amino acids found in the region. **B.** Amino acids within MOMP that donate hydrogen bonds to QPLEX have been highlighted.


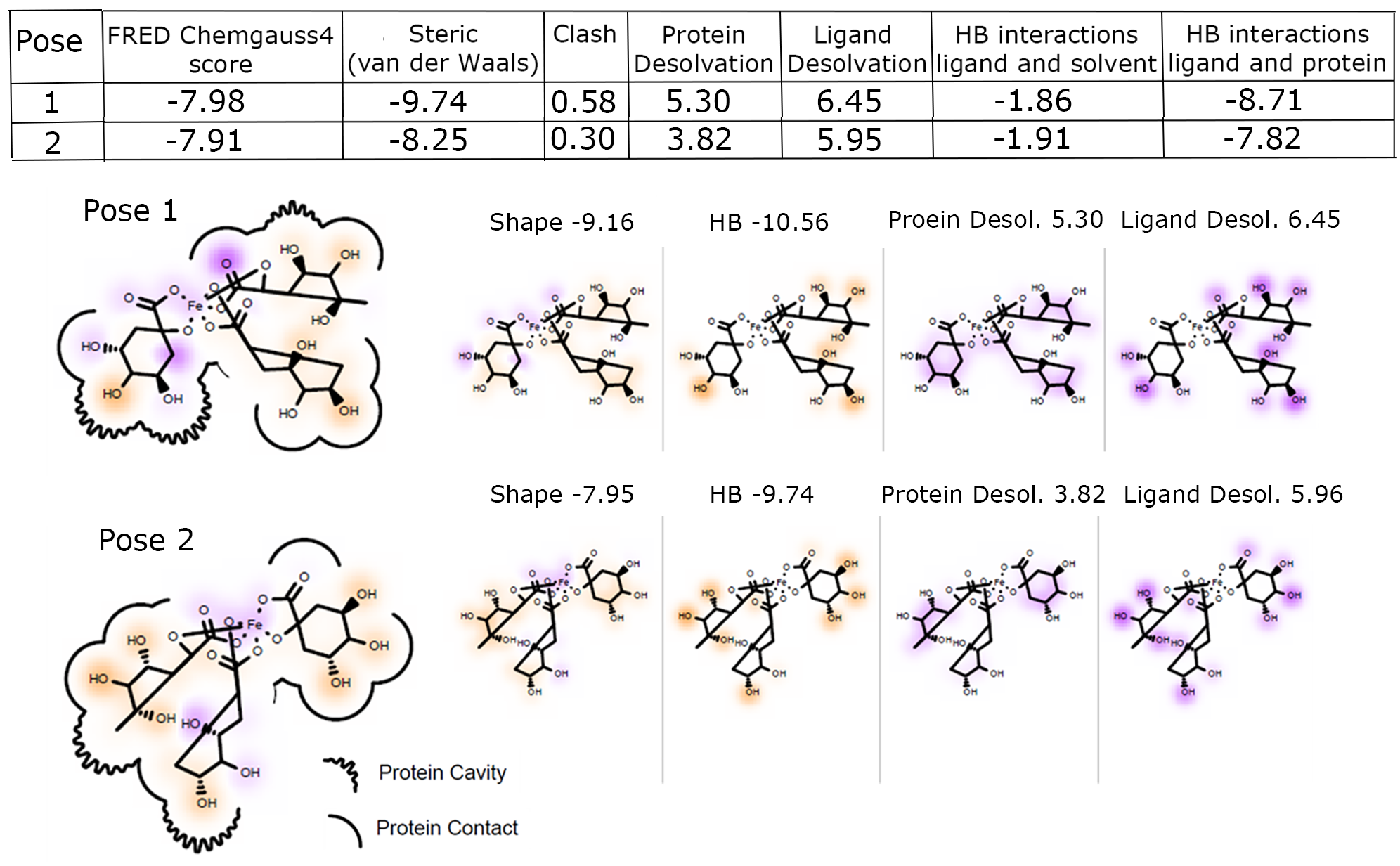


**Figure S7:** The report of the FRED Chemgauss4 score for the top two poses when docking QPLEX within the porin channel is shown at the top. The poses are ranked in order of FRED Chemgauss4 score which is a function of the steric, clash, protein desolvation, ligand desolvation scores as well as the score from hydrogen bonding interactions between QPLEX and solvent and the score from hydrogen bonding interactions between QPLEX and MOMP.

2

1


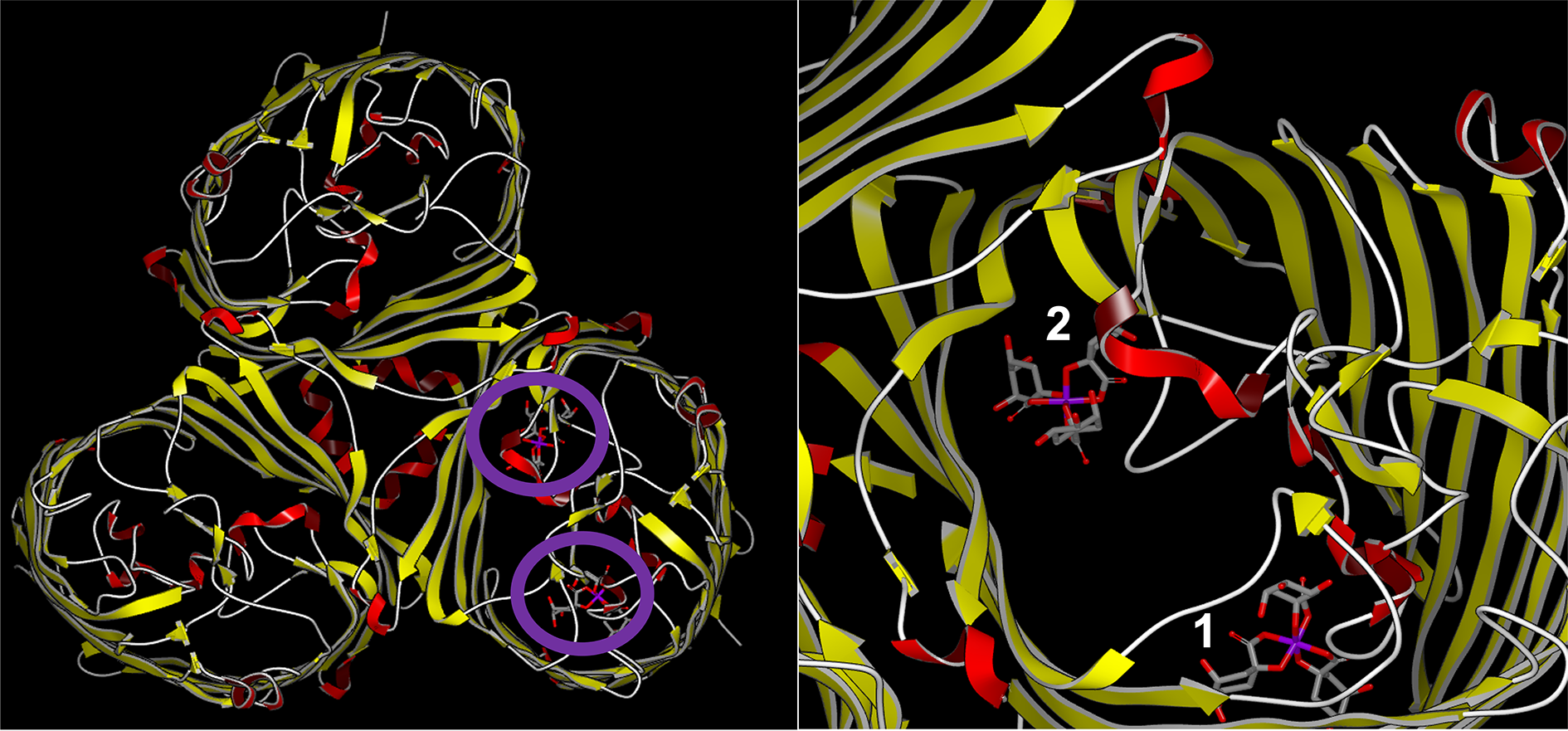


**Figure S8:** The two highest ranked poses of QPLEX docked within the porin channel. Pose 1 is closer to the surface of the protein whilst pose 2 is located deeper within the channel closer to the periplasm.


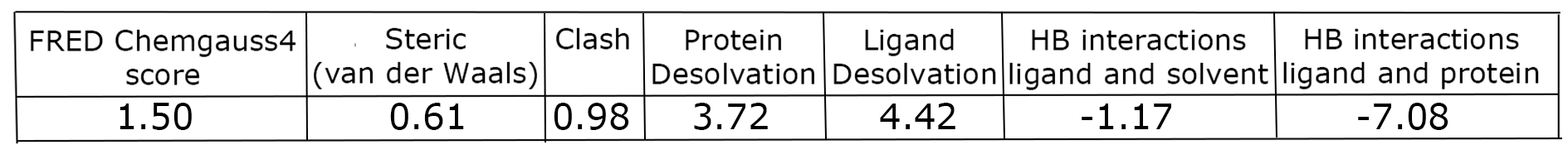


**Figure S9:** The report of the FRED Chemgauss4 score for the top pose when docking QPLEX to the intracellular portion of MOMP in triplicate experiments. Only the top ranking pose out of ten poses is shown here. The poses were ranked in order of FRED Chemgauss4 score which is a function of the steric, clash, protein desolvation, ligand desolvation scores as well as the score from hydrogen bonding interactions between QPLEX and solvent and the score from hydrogen bonding interactions between QPLEX and MOMP.

**
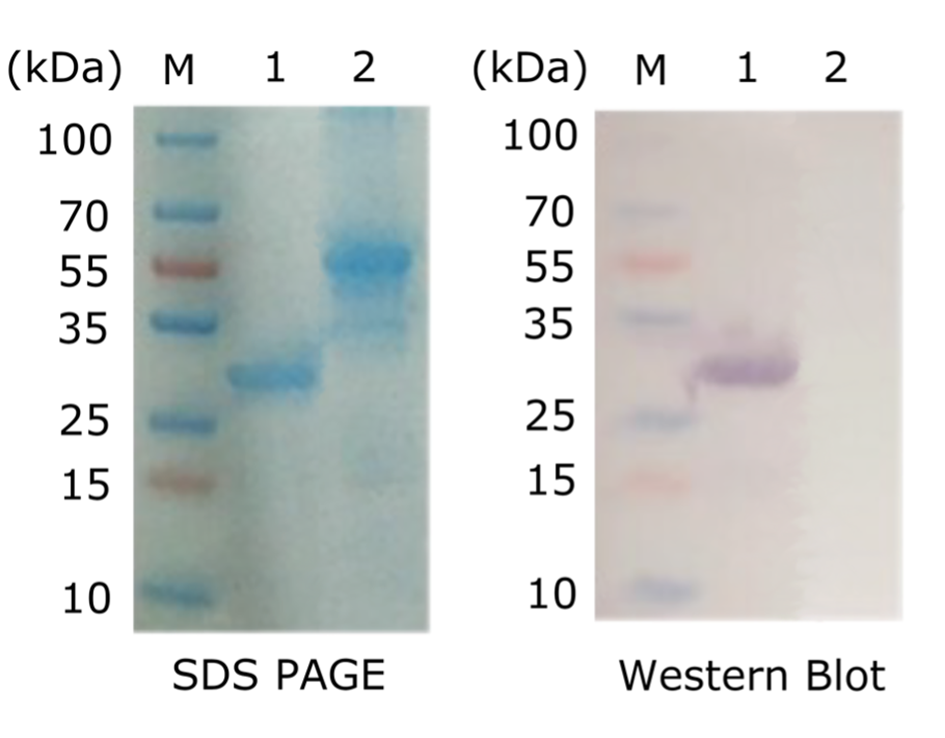
**

**Figure S10:** Western blot analysis of the MOMP NCTC11168 (lane 1) and BSA control (lane 2). Proteins were resolved through an SDS PAGE gel (left panel) and blotted onto nitrocellulose for a western blot (right panel) with rabbit anti-MOMP antibody. Lane M shows a ThermoFisher Prestained PageRuler Plus Stained Protein Ladder and lane 2 shows control BSA.

**
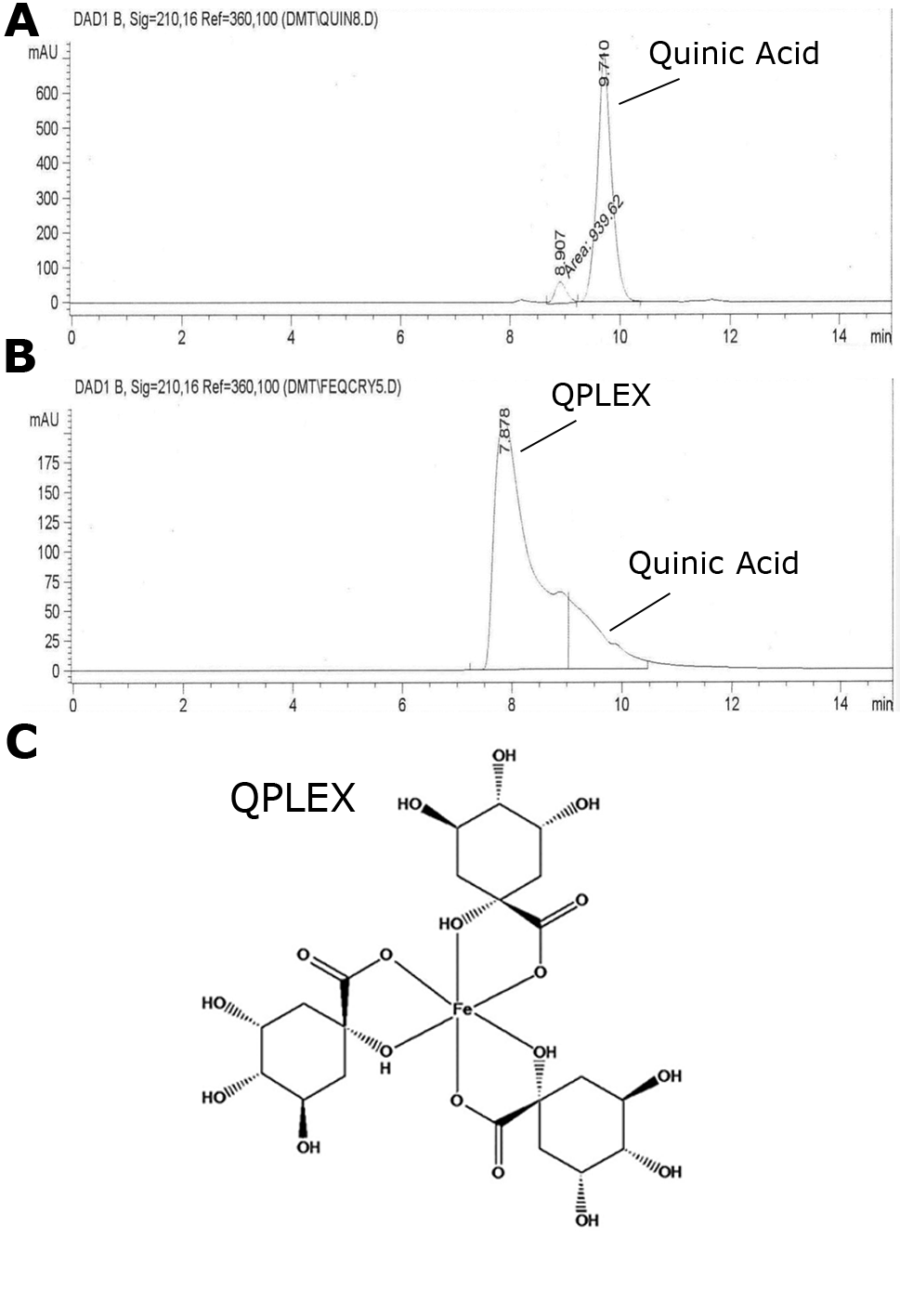
**

**Figure S11:** HPLC chromatographs of quinic acid (**A**) and QPLEX (**B**) to check the synthesis and purity of the latter. Quinic acid eluted at 9.710 minutes whereas QPLEX eluted at 7.87 minutes. The small peak at 8.907 minuts in panel **A** indicates a small impurity in the quinic acid. From integrating the area under the peak we can deduce that the purity of QPLEX was 94%. The structure of QPLEX is shown in panel **C**.

**
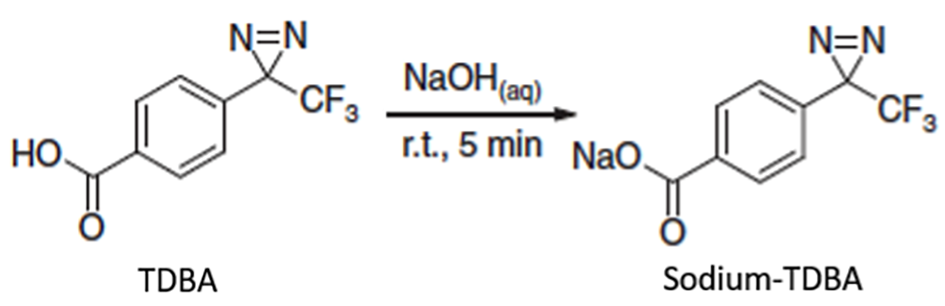
**

**Figure S12:** Synthesis of the carbene footprinting probe.

The addition of sodium hydroxide to TDBA produces the carbene. The carbene contains an aromatic ring to provide a non-polar surface for interaction with hydrophobic patches on the protein, and an ionic group both to associate with polar amino acid side chains and to improve water solubility of the probe. The CF3 group has the benefit of reducing side reactions associated with the diazoisomer.


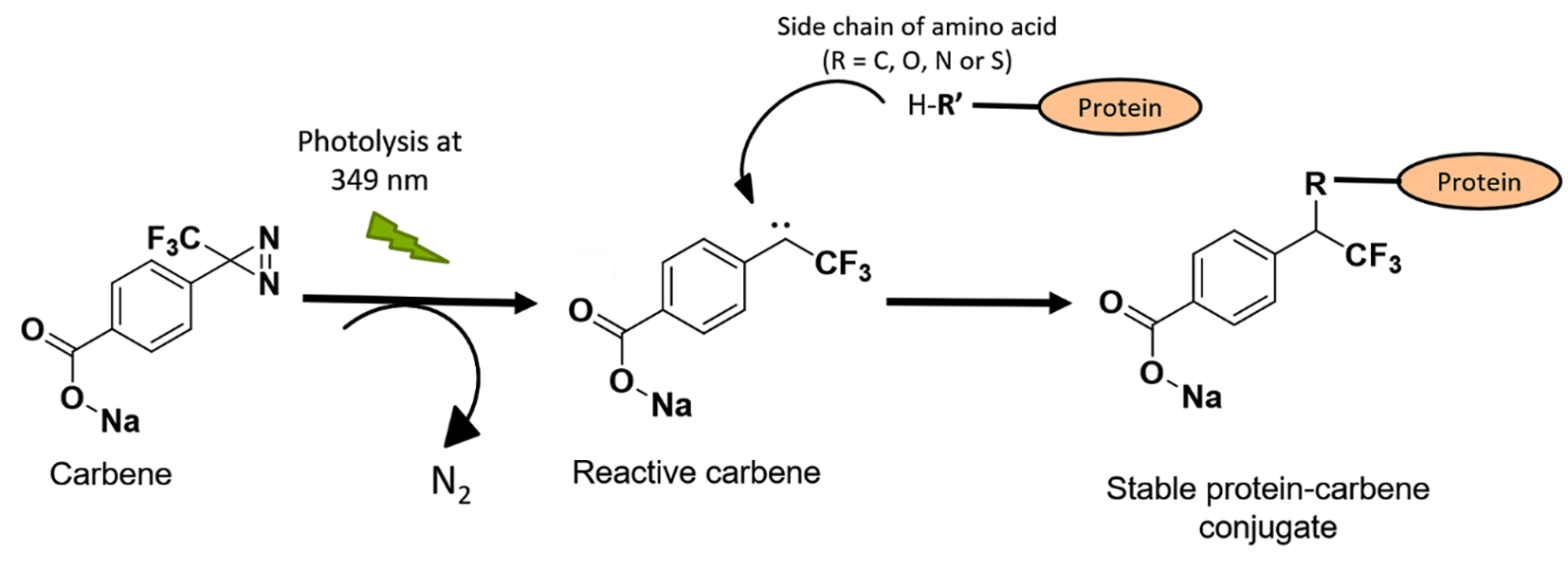


**Figure S13:** Irradiation (photolysis at near-UV laser at 349 nM) and flash freezing of the carbene has the dual benefit of halting enzyme turn-over and reducing diffusion of the probe. The removal of the azo group by UV leaves a lone pair that forms a reactive carbene with the potential to rapidly insert into any R–H bond (where R can be C, O, N, or S) within the protein’s amino acid side chains.

Peptide 23-30


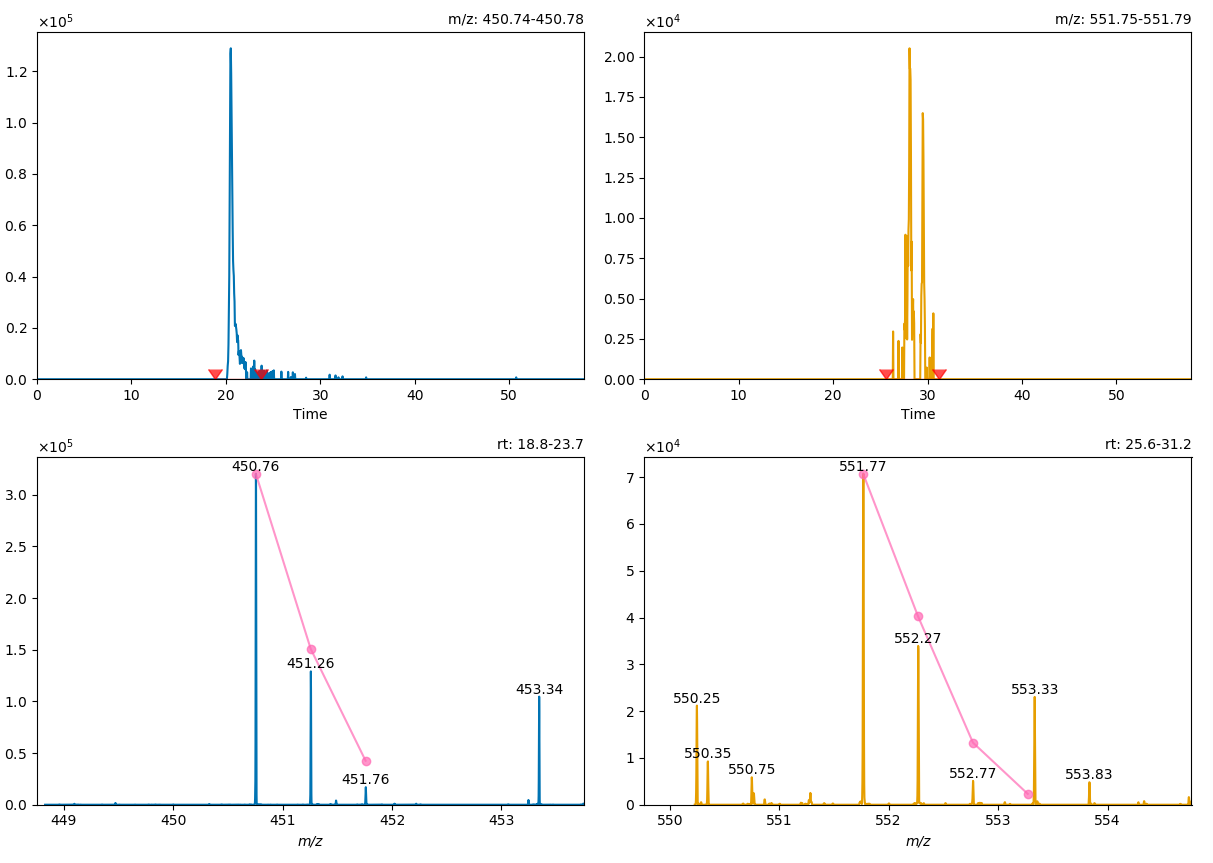


Peptide 31-39


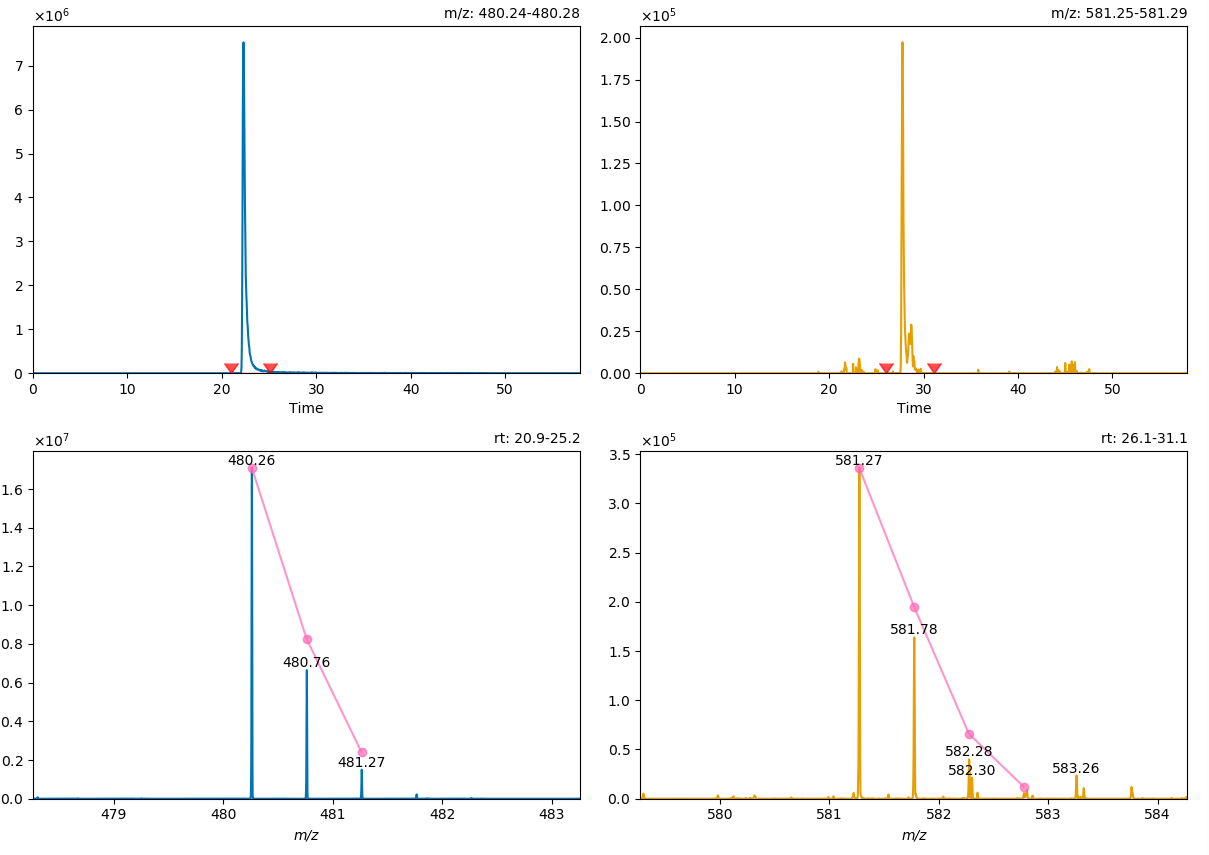


Peptide 40-49


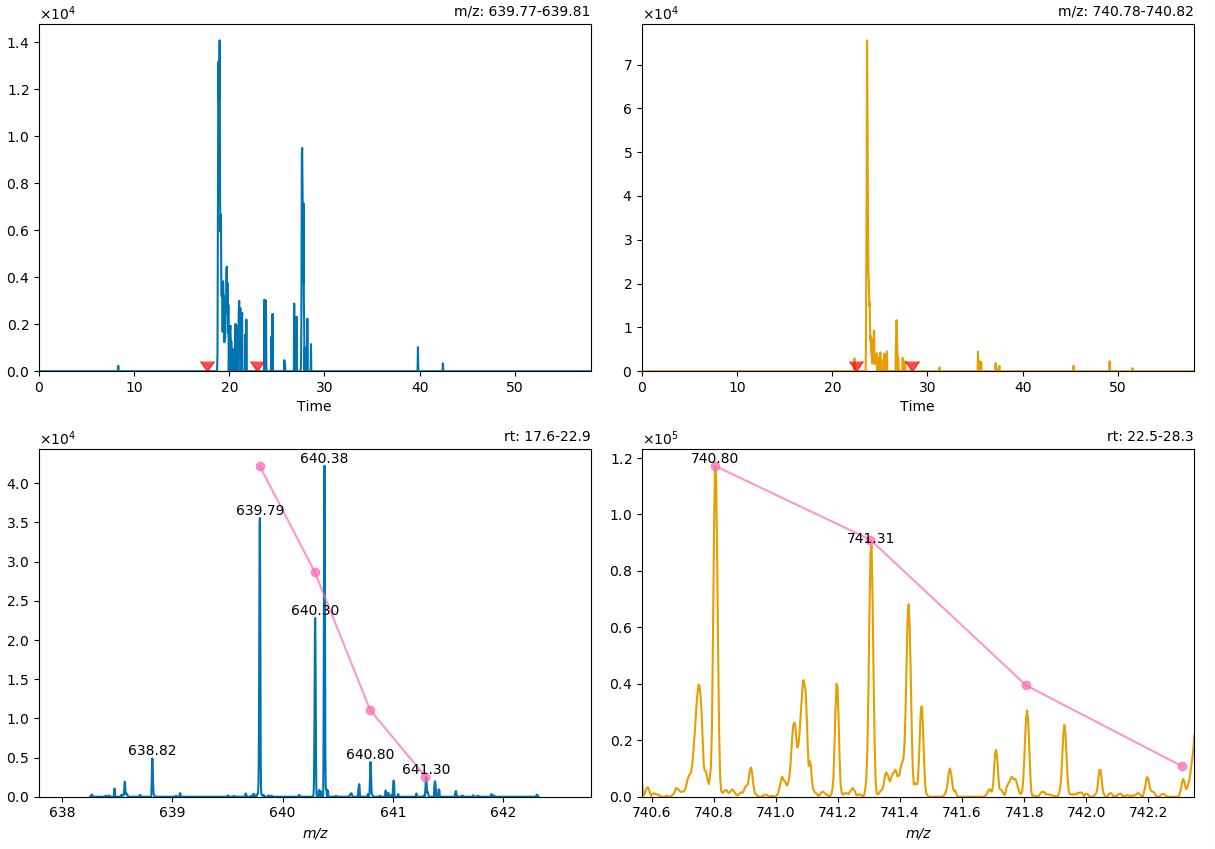


Peptide 40-61


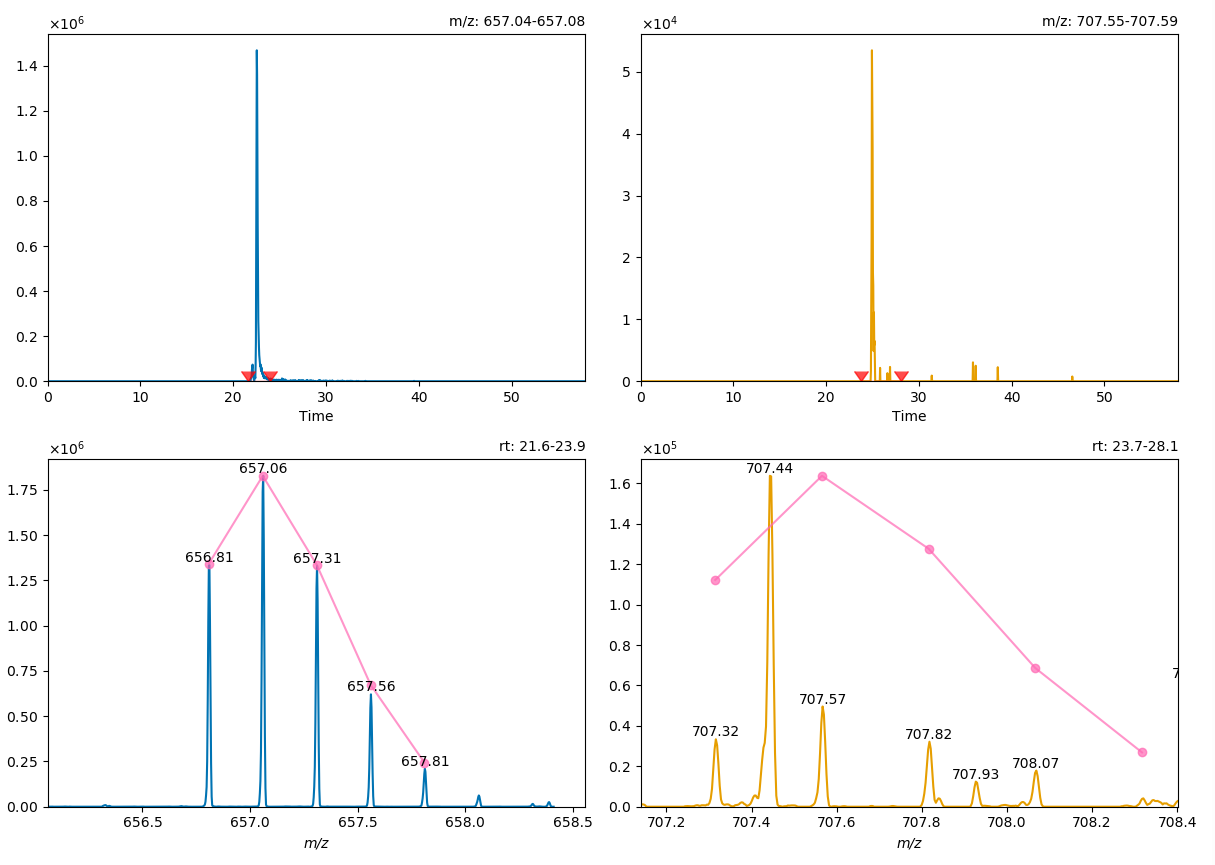


Peptide 42-49


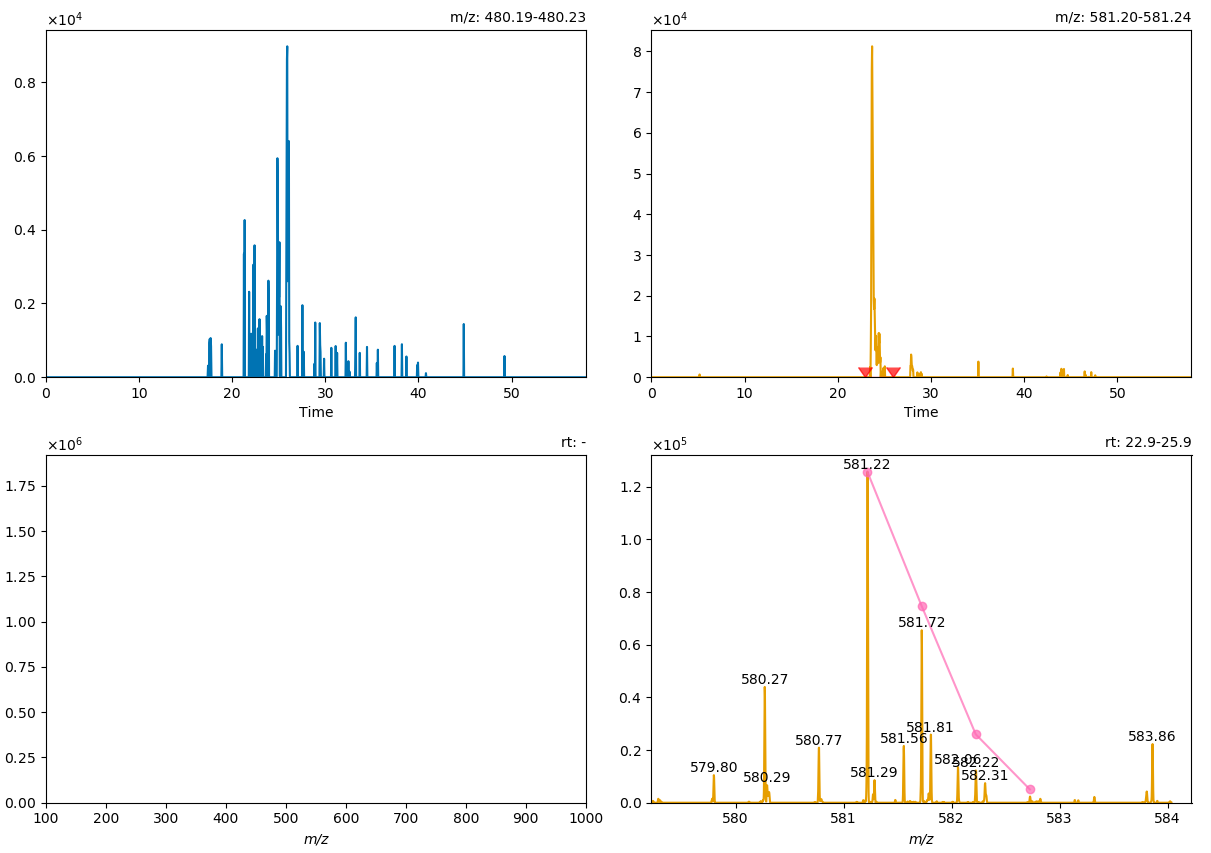


Peptide 42-61


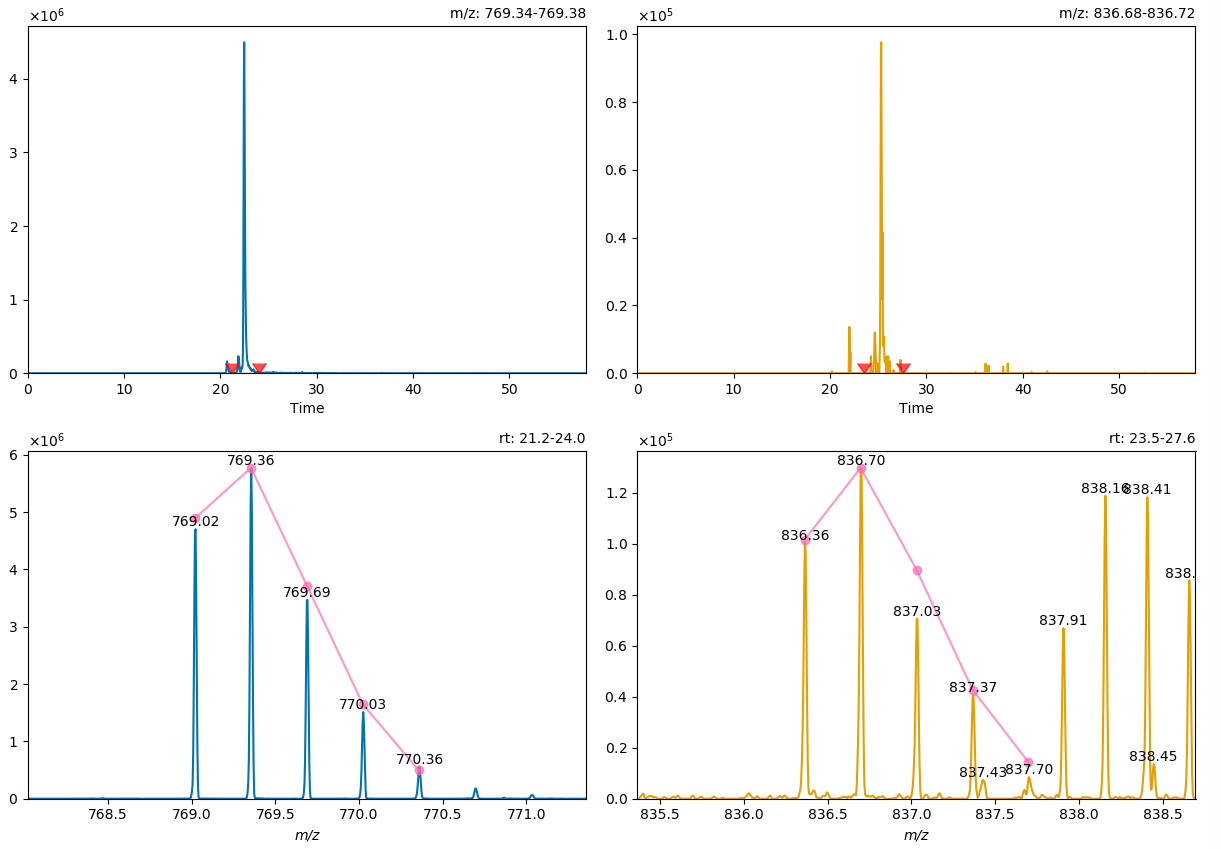


Peptide 50-61


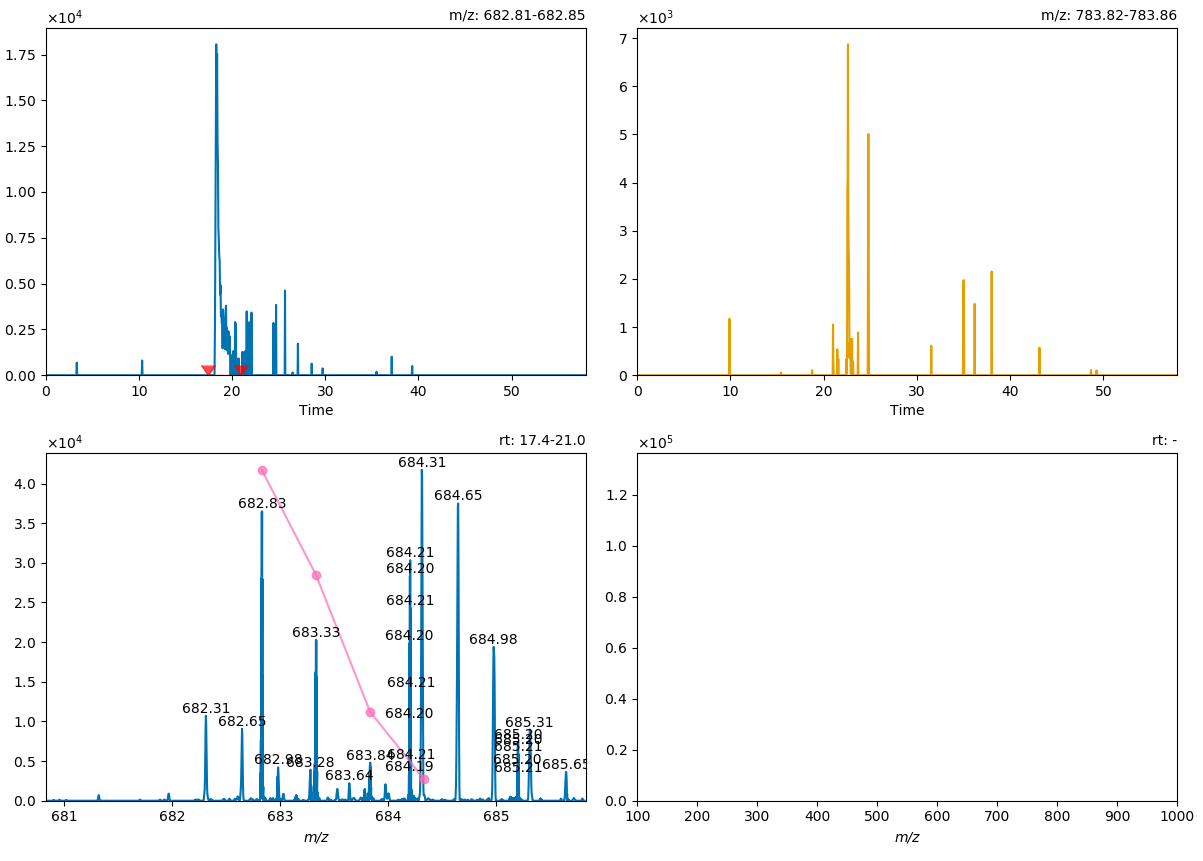


Peptide 68-81


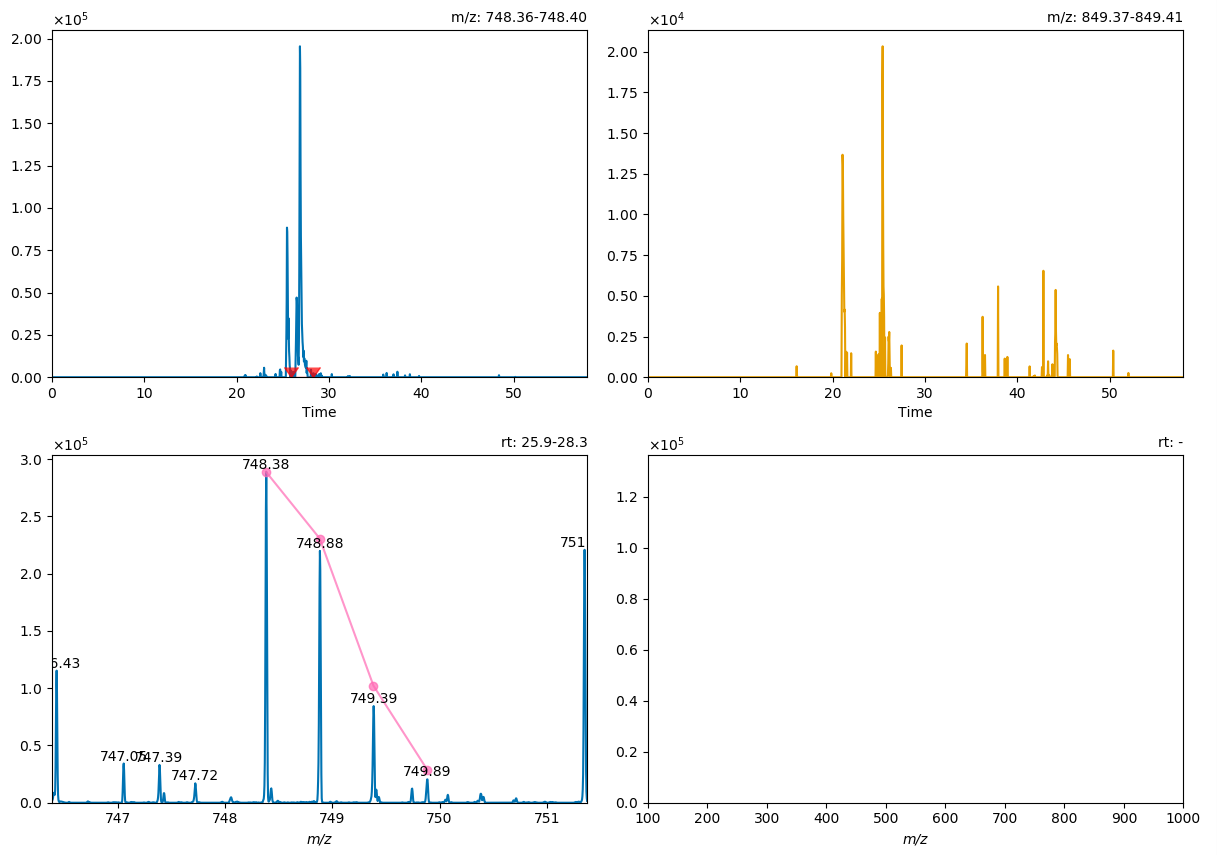


Peptide 102-110


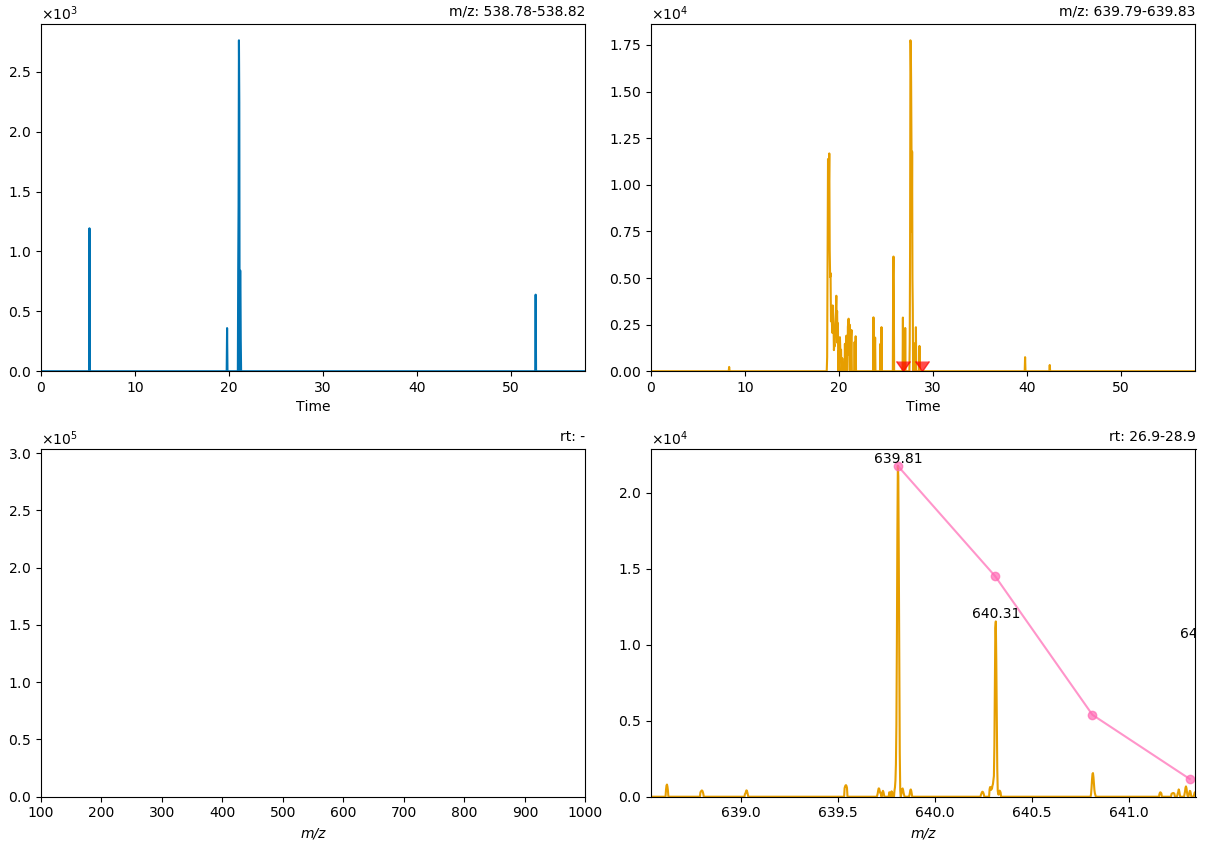


Peptide 111-129


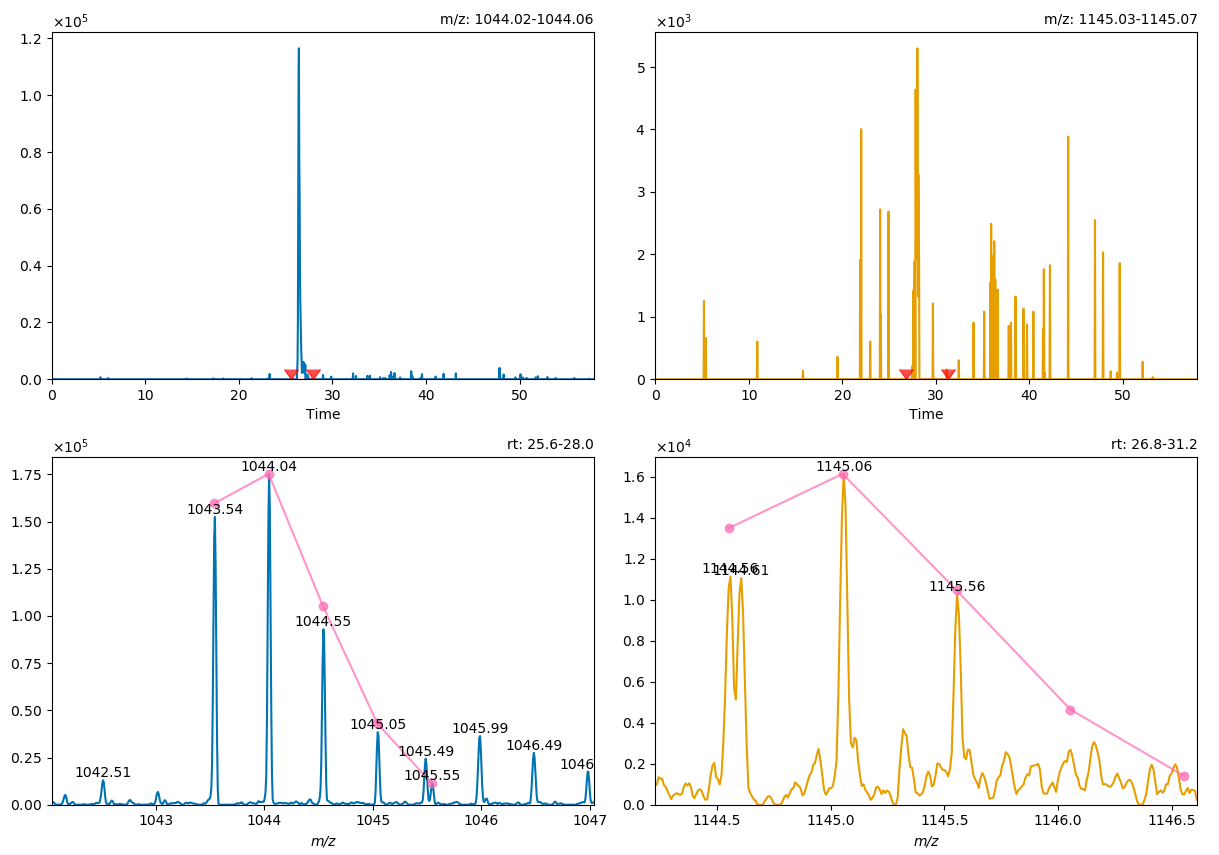


Peptide 268-278


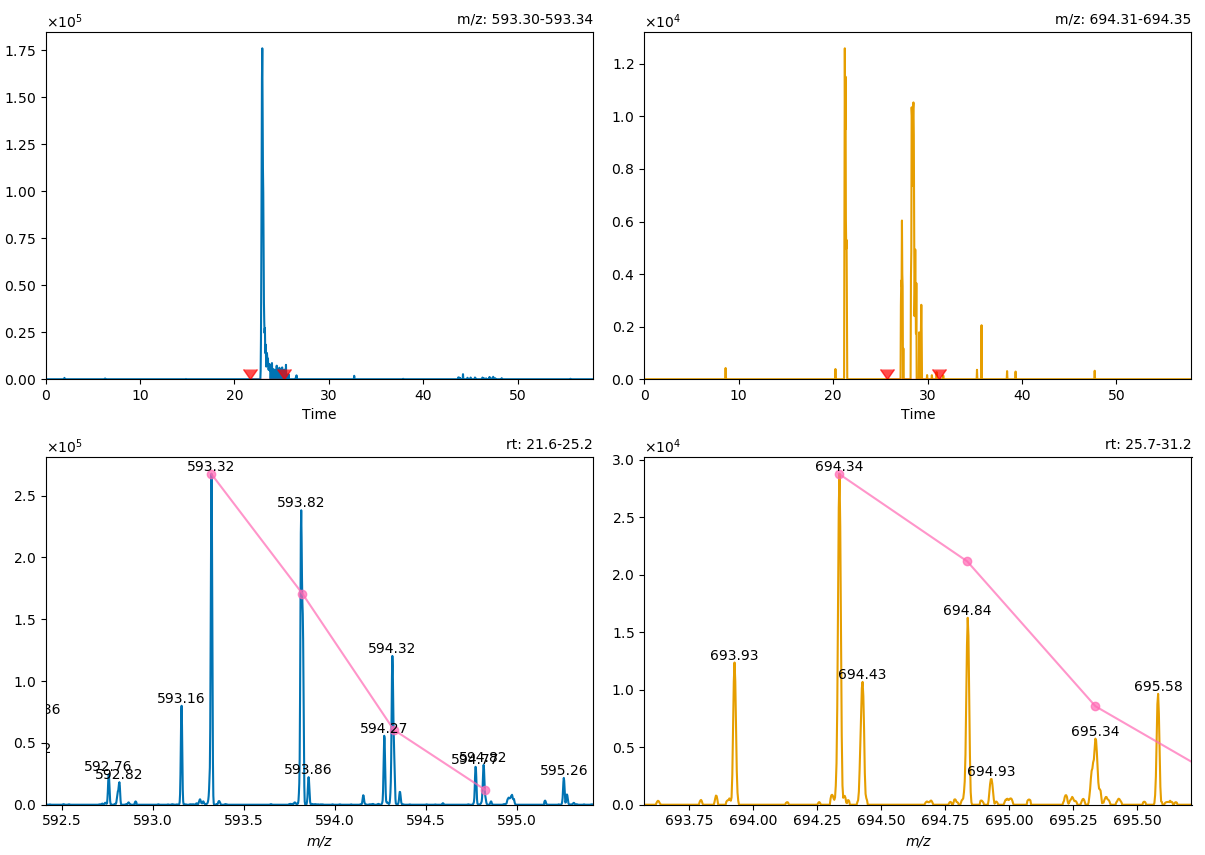


Peptide 279-298


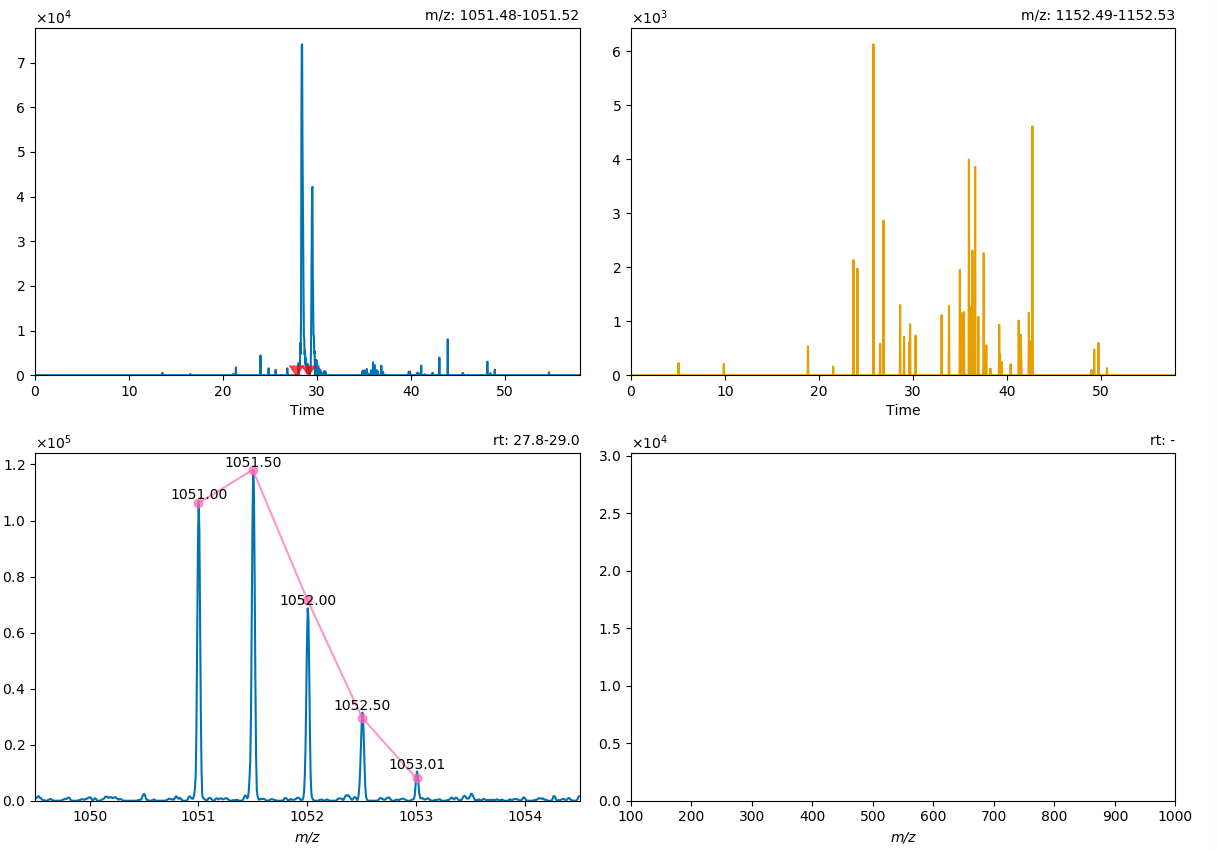


Peptide 301-328


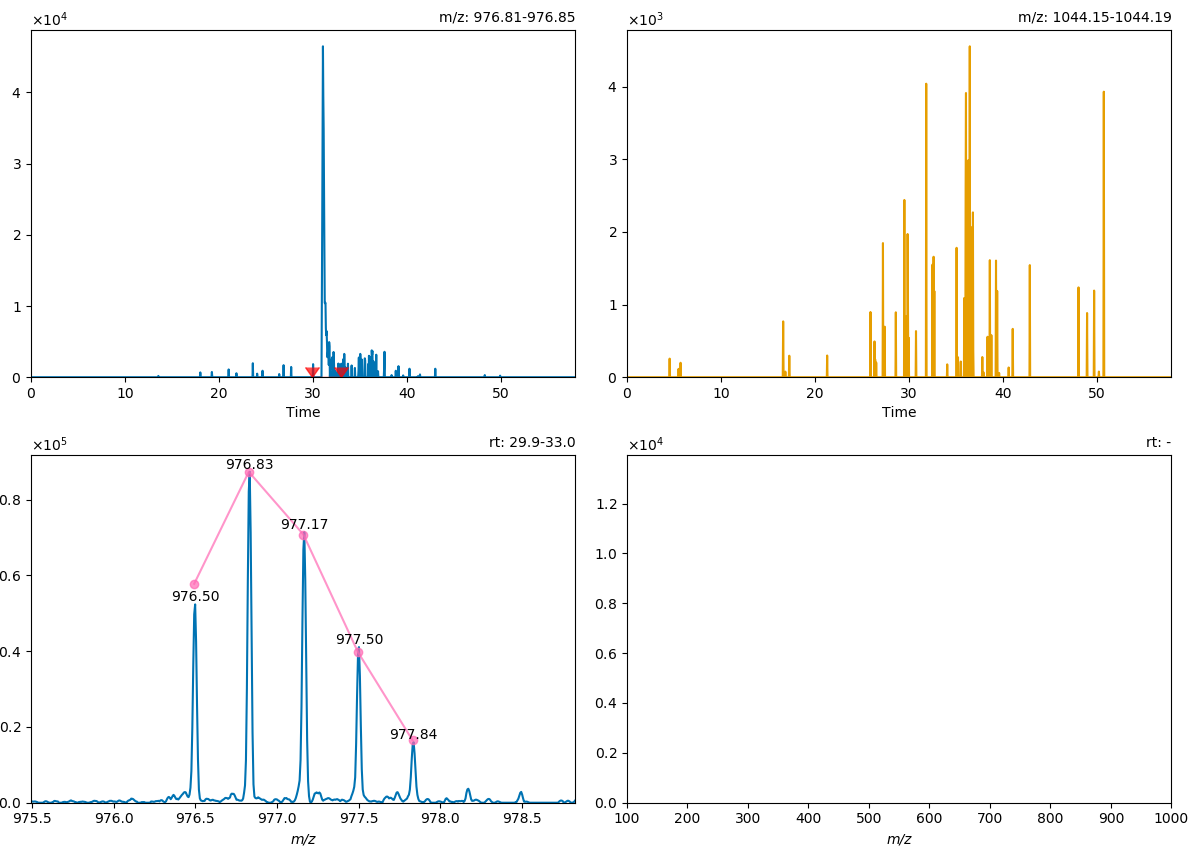


Peptide 336-352


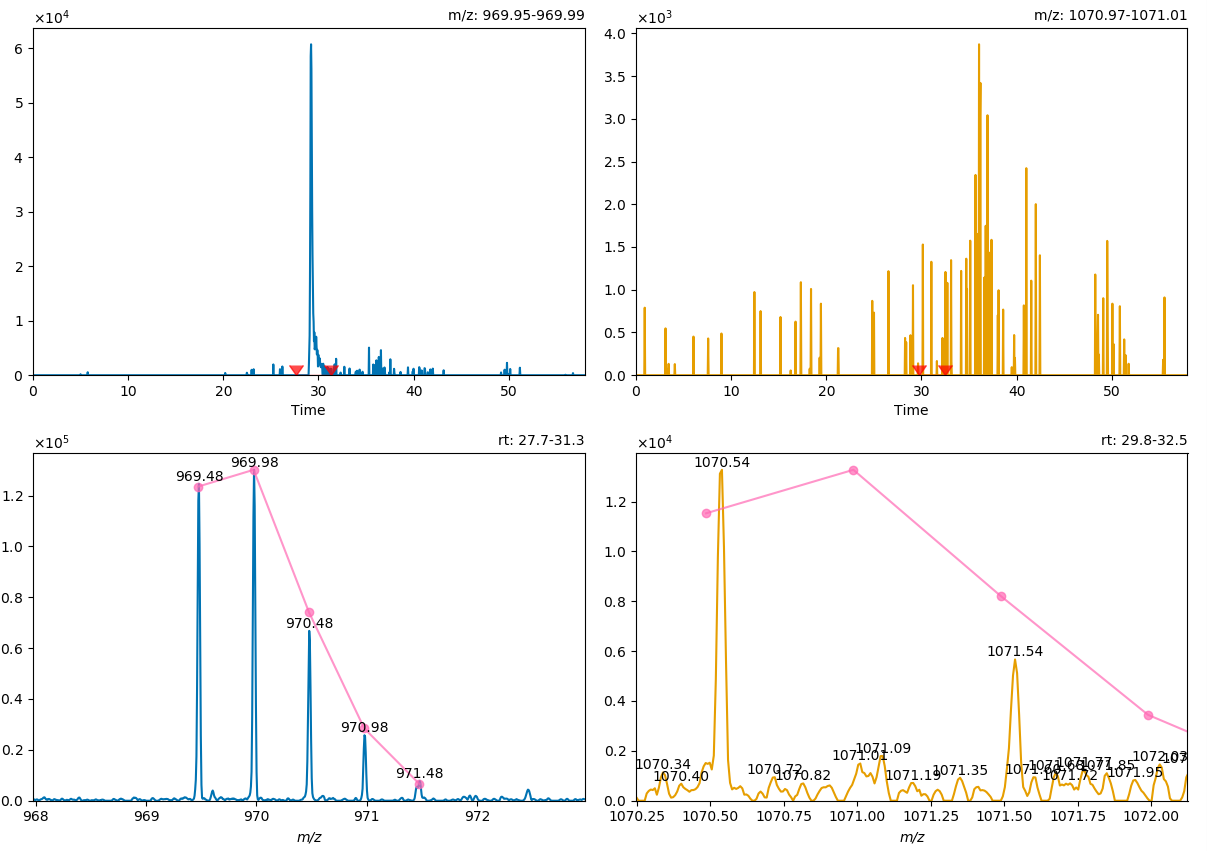


Peptide 353-363


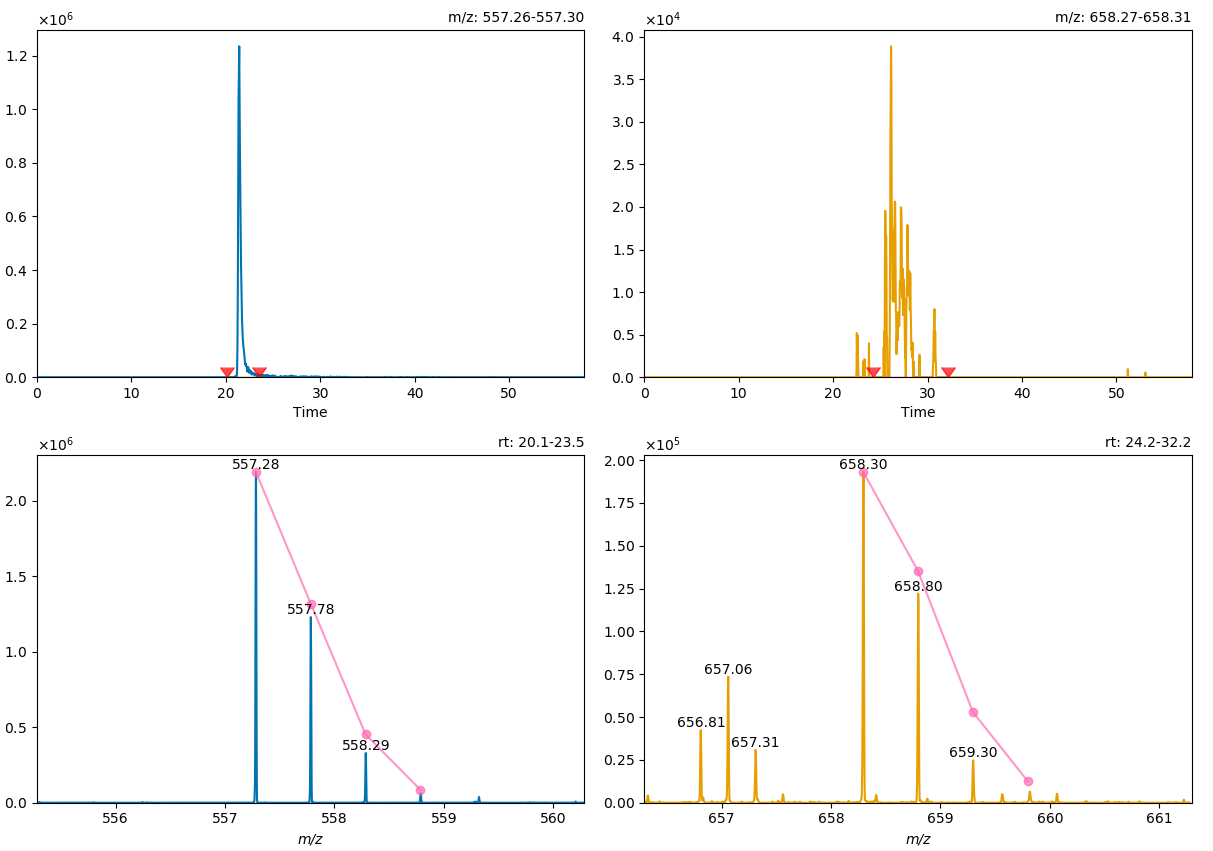


Peptide 353-374


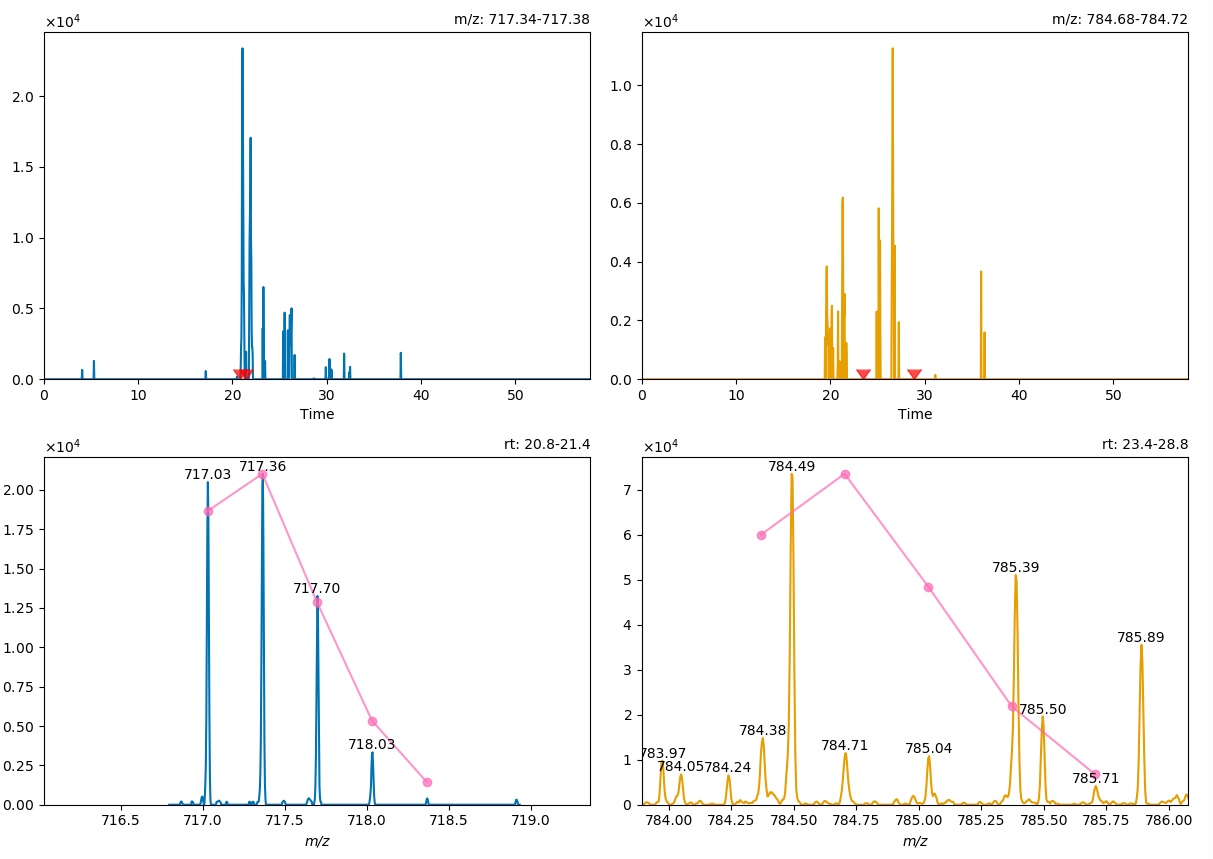


Peptide 382-389


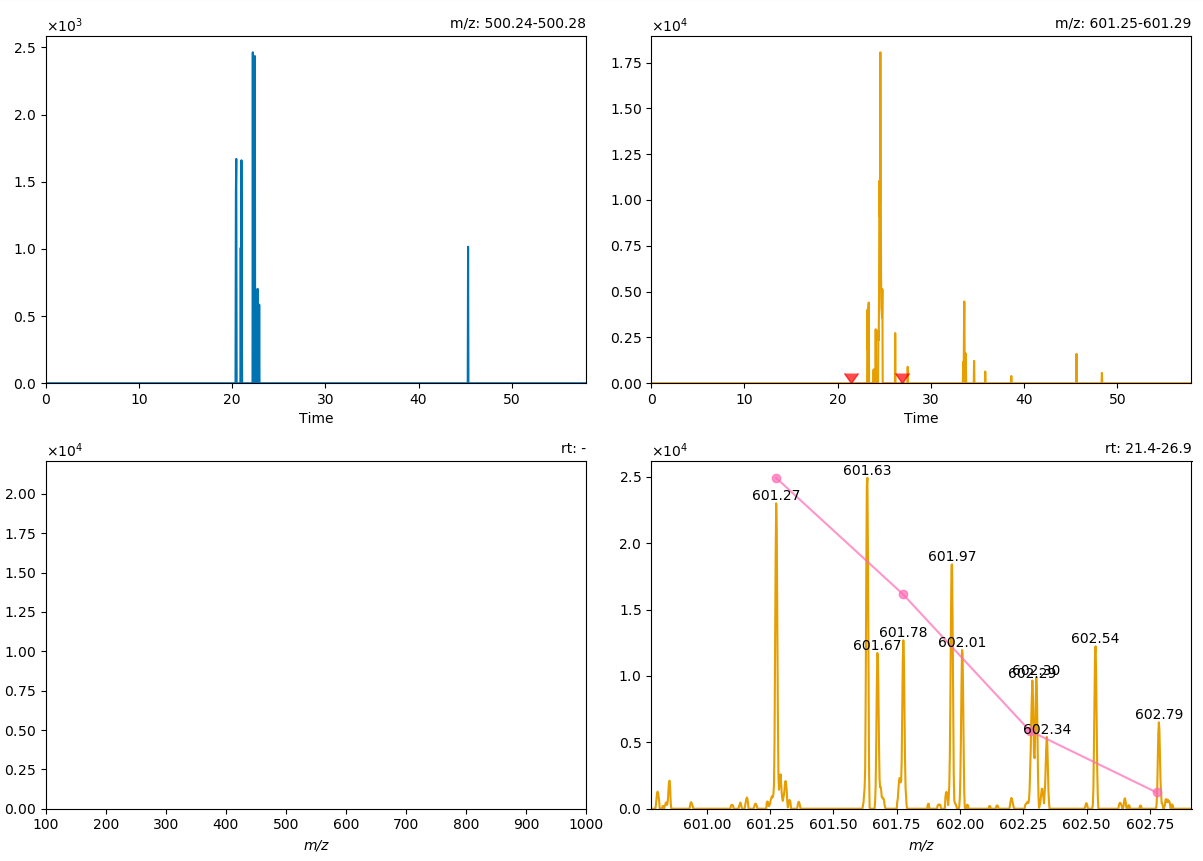


Peptide 390-417


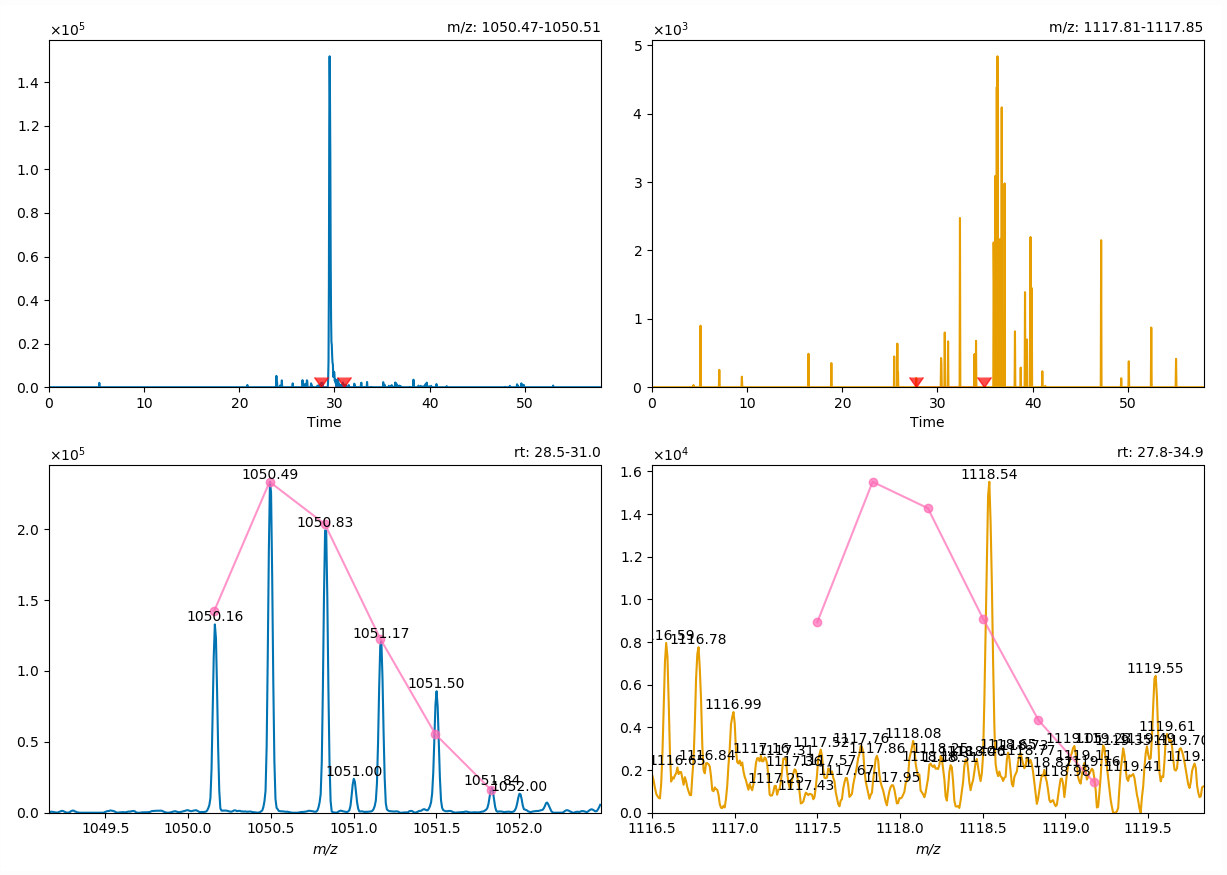


Peptide 418-423


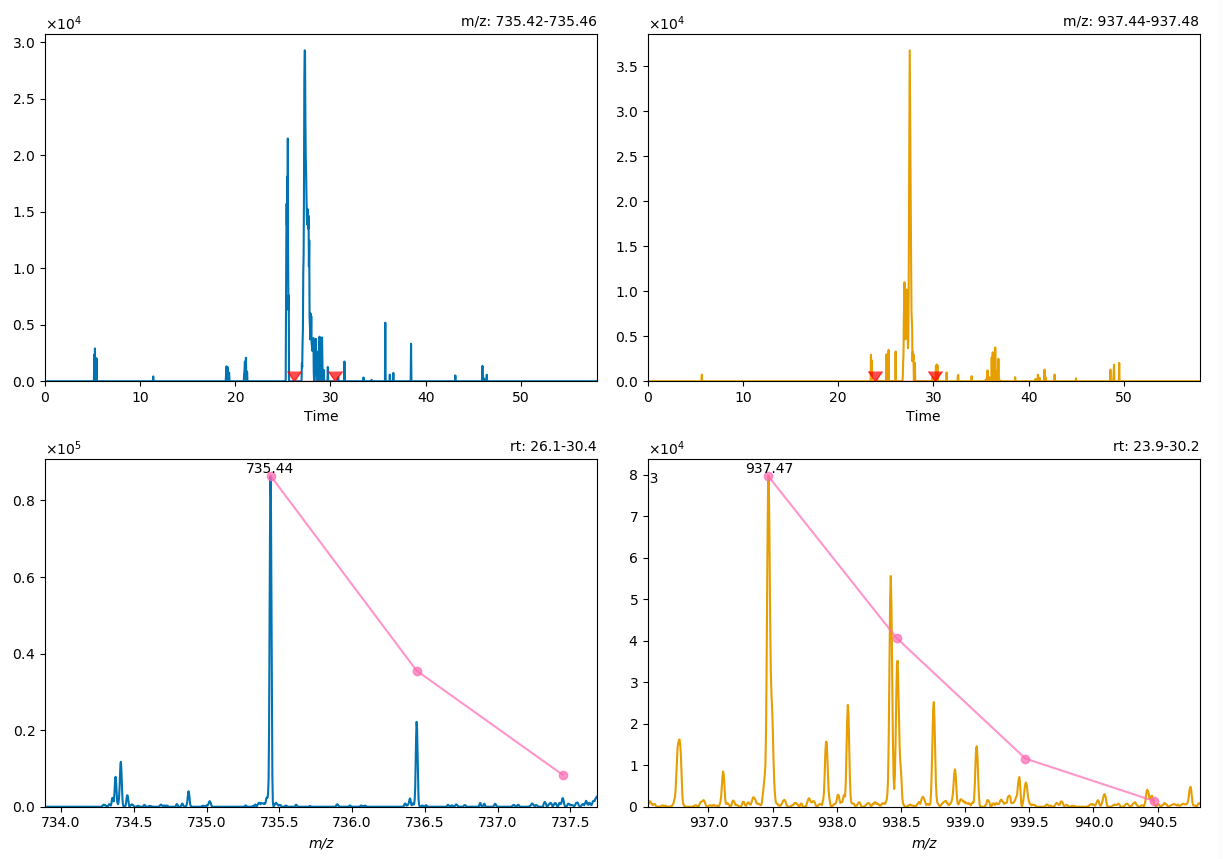


Peptide 418-424


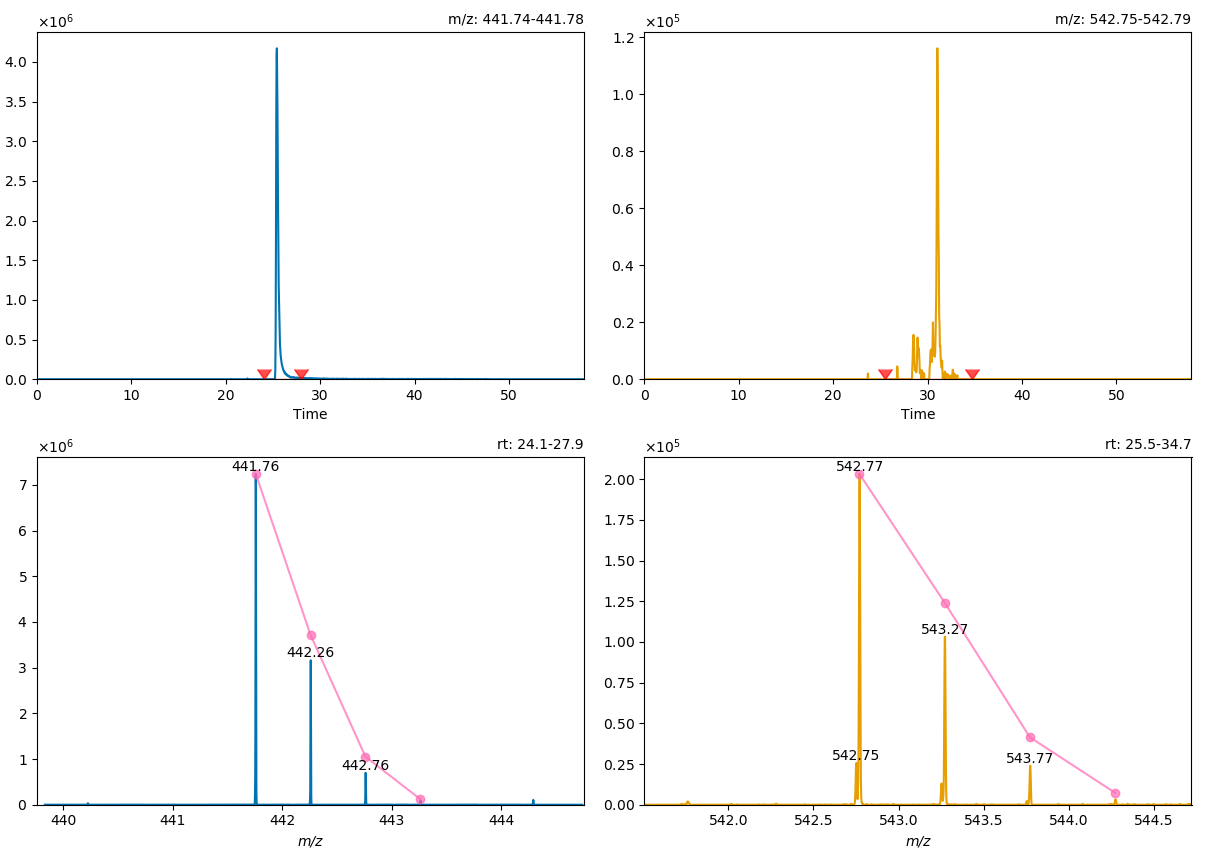


**Figure S14**. Representative PepFoot analysis of carbene labelling in MOMP (actual sample data used is from control (- QPLEX) 01). Data are shown for each tryptic peptide. Blue: extracted ion chromatogram and mass spectrum for unlabelled ion, orange: extracted ion chromatogram and mass spectrum for labelled ion.


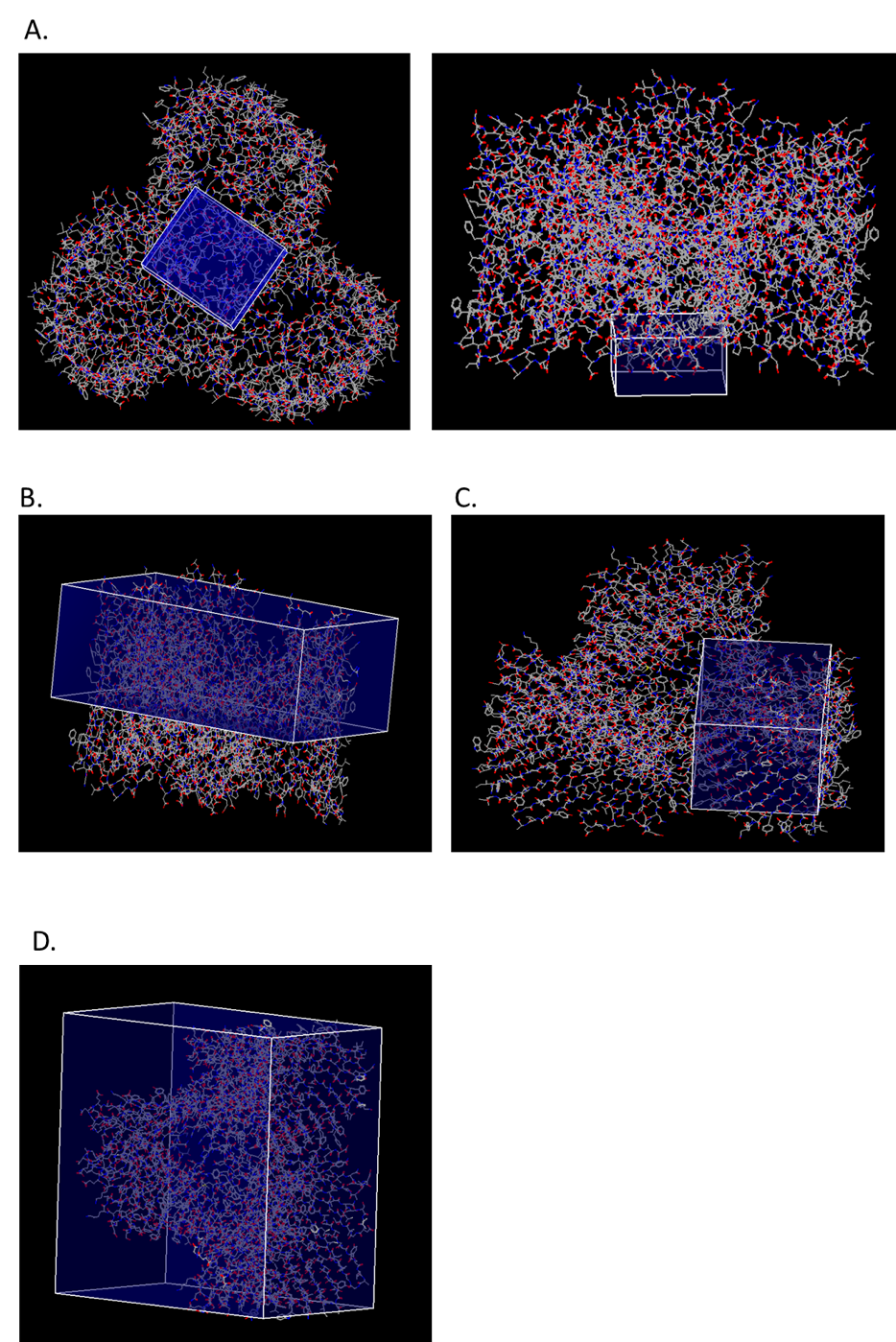


**Figure S15:** In the MakeReceptor software there are three stages before a receptor is complete; molecule, box and shape constraints. During the ‘molecule’ stage the pdb file 5LDT of MOMP 85H was loaded into the programme. Chains A, B, and C were marked as protein whilst the crystallographic waters, bound ligands and other non-bonded solvent molecules were ignored as extra molecules. In the ‘box’ stage a box was defined, enclosing the active site where heavy atoms of the docking ligand would be placed. Any docked pose with any heavy atom that lies outside the box were rejected. In the final stage, the shape of the active site, also known as ‘negative image’, was defined by the inner and outer contours of the receptor. Both contours were disabled to increase the space of poses searched for each docking ligand, although docking without the outer contour increased docking time by as much as 100-fold.
